# Genetic risk of osteoarthritis operates during human skeletogenesis

**DOI:** 10.1093/hmg/ddac251

**Published:** 2022-10-09

**Authors:** Sarah J Rice, Abby Brumwell, Julia Falk, Yulia S Kehayova, John Casement, Eleanor Parker, Ines M J Hofer, Colin Shepherd, John Loughlin

**Affiliations:** Biosciences Institute, International Centre for Life, Newcastle University, Newcastle upon Tyne NE1 3BZ, UK; Biosciences Institute, International Centre for Life, Newcastle University, Newcastle upon Tyne NE1 3BZ, UK; Biosciences Institute, International Centre for Life, Newcastle University, Newcastle upon Tyne NE1 3BZ, UK; Biosciences Institute, International Centre for Life, Newcastle University, Newcastle upon Tyne NE1 3BZ, UK; Bioinformatics Support Unit, Faculty of Medical Sciences, Newcastle University, Framlington Place, Newcastle upon Tyne NE2 4HH, UK; Biosciences Institute, International Centre for Life, Newcastle University, Newcastle upon Tyne NE1 3BZ, UK; Biosciences Institute, International Centre for Life, Newcastle University, Newcastle upon Tyne NE1 3BZ, UK; Biosciences Institute, International Centre for Life, Newcastle University, Newcastle upon Tyne NE1 3BZ, UK; Biosciences Institute, International Centre for Life, Newcastle University, Newcastle upon Tyne NE1 3BZ, UK

## Abstract

Osteoarthritis (OA) is a polygenic disease of older people resulting in the breakdown of cartilage within articular joints. Although it is a leading cause of disability, there are no disease-modifying therapies. Evidence is emerging to support the origins of OA in skeletogenesis. Whereas methylation quantitative trait loci (mQTLs) co-localizing with OA genome-wide association study signals have been identified in aged human cartilage and used to identify effector genes and variants, such analyses have never been conducted during human development. Here, for the first time, we have investigated the developmental origins of OA genetic risk at seven well-characterized OA risk loci, comprising 39 OA-mQTL CpGs, in human fetal limb (FL) and cartilage (FC) tissues using a range of molecular genetic techniques. We identified significant OA-mQTLs at 14 and 29 CpGs in FL and FC tissues, respectively, and compared our results with aged cartilage samples (AC). Differential methylation was observed at 26 sites between FC and AC, with the majority becoming actively hypermethylated in old age. Notably, 6/9 OA effector genes showed allelic expression imbalances during fetal development. Finally, we conducted ATAC-sequencing in cartilage from the developing and aged hip and knee to identify accessible chromatin regions and found enrichment for transcription factor binding motifs including SOX9 and FOS/JUN. For the first time, we have demonstrated the activity of OA-mQTLs and expression imbalance of OA effector genes during human skeletogenesis. We show striking differences in the spatiotemporal function of these loci, contributing to our understanding of OA aetiology, with implications for the timing and strategy of pharmacological interventions.

## Graphical Abstract

**Graphical Abstract:**
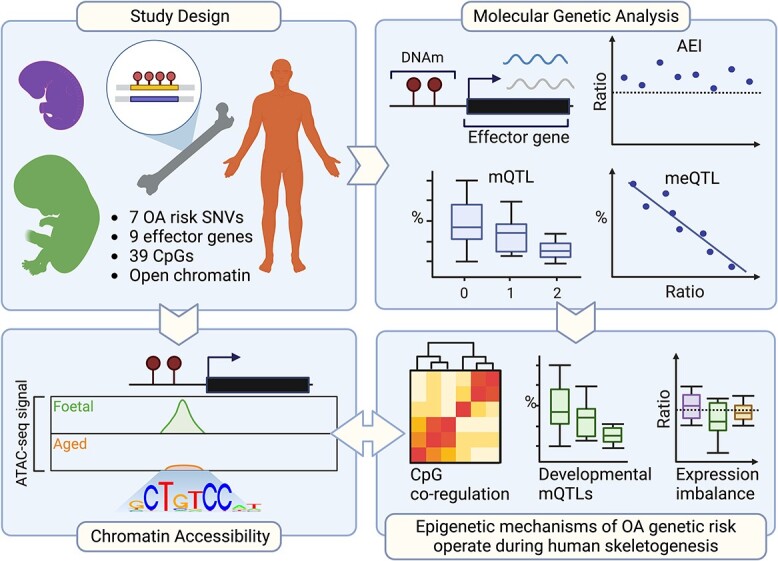

## Introduction

Osteoarthritis (OA) is a complex disease of the articulating joints, typically impacting those over the age of 45, with the risk of developing progressive disease increasing with age (1). OA impacts the lives of over 500 million individuals worldwide, a figure that increased by 48% between 1990 and 2019, and which continues to rise in ageing populations (2). Despite this, there are currently no disease-modifying OA drugs (DMOADs) available.

In the healthy joint, the articular cartilage lubricates joint surfaces and absorbs mechanical impact. In OA, this cartilaginous surface roughens and breaks down, eventually exposing the underlying bone. This leads to chronic pain, disability and premature death from secondary co-morbidities (3,4). The aetiology of OA is complex and multifactorial, yet genetic variation accounts for up to 50% of the total disease risk, placing it as one of the highest single risk factors (1,5).

The developmental origins of OA have long been debated due to a plethora of factors including the association of disease with abnormal joint morphology, the extent of disease heritability (6–8) and the reversion to a developmental phenotype within hypertrophic OA articular chondrocytes (9,10). The articular chondrocyte, the sole cell type in cartilage, undergoes very little proliferation once adulthood is reached, with the non-dividing cells remaining metabolically active. This raises the question of whether defects in cartilage integrity conferred by genetic variation are established during embryonic and fetal development, manifesting in later life (10). Such evidence would have enormous implications upon the successful development of DMOADs and the determination of a ‘window of opportunity’ for pharmacological intervention in early-stage disease (11). As a result, OA research has heavily utilized small animal developmental models, along with stem cell models of chondrogenesis (12,13), and, more recently, human fetal samples (8).

Over 100 common genetic variants, namely single nucleotide variants (SNVs), have been reported, which reproducibly associate with OA genetic risk (14). Identified through genome-wide association studies (GWAS), the reported SNVs are overwhelmingly enriched within non-coding regions of the genome, and often fall in regions of high linkage disequilibrium (LD), whereby variant alleles are co-inherited, making it difficult to identify causal variants and effector genes at the loci.

Across the field of complex disease research, the identification of methylation and expression quantitative trait loci (mQTLs and eQTLs, respectively) is increasingly being applied to prioritize candidate variants and genes at disease risk loci (15–17). Furthermore, in studies of neuropsychiatric disorders, disease-associated mQTLs have been identified in developing fetal brain tissue, predisposing individuals to disease from the start of life (18). In OA, the identification of mQTLs and eQTLs (the latter often discovered by allelic expression imbalance, AEI, analysis) has been applied to prioritize causal SNVs, effector genes and regulatory elements in aged joint tissues (14,19–23). Successful follow-up studies using targeted epigenome editing have demonstrated causal effects, and functional roles of DNA methylation (DNAm) in altering the expression of effector genes (22,23). However, such QTL analyses have not previously been applied to relevant human fetal tissues to investigate developmental origins of musculoskeletal disease.

In this study, we investigated seven OA genetic risk loci distributed across the human genome, at which mQTLs at 39 CpGs have previously been identified and replicated in aged human cartilage (19–22,24–26). We hypothesized that the epigenetic mechanisms of gene regulation found in the aged human cartilage may be active during fetal development, impacting cartilage integrity from the beginning of life. We quantified DNAm at these CpGs in pooled limb tissues from individual human fetuses and in fetal joint cartilage. Further, we conducted a series of molecular genetic analyses to investigate allele specific gene expression in developmental samples. Finally, we conducted assay for transposase accessible chromatin sequencing (ATAC-seq) in developmental and aged hip and knee cartilage to compare the chromatin accessibility between development and in OA.

## Results

### DNAm profiling of FL tissues at OA risk loci

We extracted DNA from two human developmental tissue types: whole limb tissues, which had a mean developmental age of 56 days (E56), and isolated limb cartilage samples, which were significantly older (E86; *P* = 5.4 × 10^−10^) (Supplementary Material, Fig. S1A and B). For cartilage analysis, we used the cartilaginous (non-ossified) tissue from the ends of the developing long bones (Supplementary Material, Fig. S1C and D), the size of which decreased as the developing bone ossified, and which showed significantly higher expression of the cartilage marker genes *COL2A1, ACAN* and *MATN3* than the pooled limb samples (Supplementary Material, Fig. S1E). There was a significant increase in the expression of *COL2A1* and *MATN3* (both *P* = 0.01) with developmental age (Supplementary Material, Fig. S1F).

**Figure 1:**
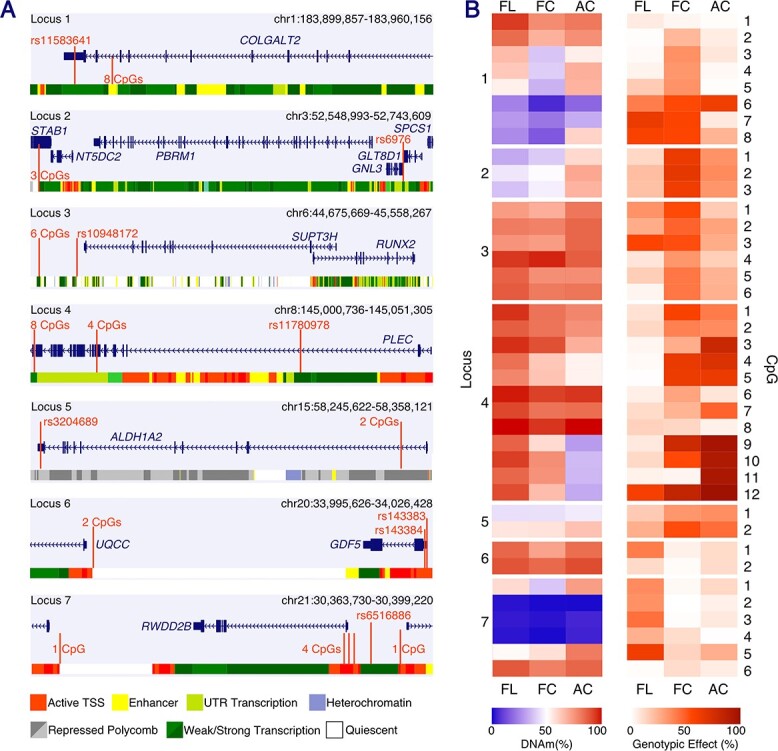
Measurements of DNAm at the 39 investigated CpGs, spanning seven OA risk loci. (**A**) Schematic diagram of the seven investigated loci showing the genomic position of each of the 39 CpGs and the OA association SNVs marking the regions. Gene transcripts are shown in dark blue. The chromatin state, determined in MSC-derived cultured chondrocytes (E049) by ChIP-seq in the ROADMAP epigenomics database, is indicated at the foot of each panel. TSS, transcription start site; UTR, untranslated region. (**B**) Left-hand panel, heatmap indicating the mean DNAm levels in each of the three investigated human tissue types: FL; FC; AC. Right-hand panel, heatmap indicating the GE, the amount by which observed changes in DNAm can be explained by the carriage of one or more risk allele, at each CpG. Both heatmaps in B–C are clustered by locus and CpG.

DNAm was quantified in both developmental tissue types at 39 OA-CpGs: sites of, or adjacent to, OA-mQTLs that have been characterized in aged cartilage. The CpGs span seven genomic loci that harbor nine OA risk genes: *COLGALT2* (Locus 1), *GNL3* and *SPCS1* (Locus 2), *SUPT3H* and *RUNX2* (Locus 3), *PLEC* (Locus 4), *ALDH1A2* (Locus 5), *GDF5* (Locus 6) and *RWDD2B* (Locus 7) (Table 1, Fig. 1A). We first compared mean DNAm at each of the CpGs in the two tissue types, fetal limb (FL, *n* = 15–19) and fetal cartilage (FC, *n* = 54–75), with DNAm levels that we had previously measured and reported in aged articular cartilage (19,21,22,24–26) (AC, *n* = 71–139) (Fig. 1B, Supplementary Material, Table S1). DNAm was significantly different between FL and FC tissues at 17/39 CpGs (adjusted *P*-value, *P*_adj_ < 0.014), highlighting the tissue-specificity of DNAm patterns during musculoskeletal development (Fig. 1B, Supplementary Material, Table S2). This was particularly striking at Locus 4, harboring *PLEC*, where DNA hypermethylation (mean > 80%) was observed in 10 of the 12 investigated CpGs in FL samples (Fig. 1B); yet in FC samples, the mean DNAm within the tissue was up to 28.8% lower (CpG 7, Supplementary Material, Table S2). When comparing the FC and AC samples, differentially methylated sites (DMS) were identified at 26/39 CpGs (Supplementary Material, Table S2), the majority (69%) demonstrating increased methylation in the aged samples. The largest differences in mean DNAm were again observed at Locus 4 (−44.36% [−47.81, −40.90]) and additionally at Locus 1, harboring *COLGALT2* (24.88% [21.44, 28.32]).

**Table 1:** Summary of the loci included in this investigation

Locus	GWAS SNP	NEA/ EA	EAF	SNP Chromosome	SNP Position	Locus CpG	Discovery CpG(s)	CpG Position (hg19)	Discovery P-value	Gene^*^	Region Annotation	References
1	rs11583641	T/C	0.83	1	183 906 245	1		183 911 970		*COLGALT2*	Intronic; enhancer	20,22
						2		183 911 999				
						3		183 912 014				
						4		183 912 020				
						5		183 912 154				
						6		183 912 187				
						7	cg18131582	183 912 305	0.003			
						8		183 912 397				
2	rs6976	C/T	0.41	3	52 728 804	1	cg18099408	52 552 593	3.73 × 10^−6^	*GNL3* *SPCS1*	Exonic	19,27
						2		52 552 598				
						3		52 552 602				
3	rs10948172	A/G	0.20	6	44 777 691	1	cg13979708	44 695 318	6.2 × 10^−5^	*SUPT3H RUNX2*	Intergenic	19,26
						2	cg19254793	44 695 348	9.00 × 10^−03^			
						3	cg20913747	44 695 427	4.9 × 10^−12^			
						4	cg18551225	44 695 536	1.12 × 10^−10^			
						5		44 695 543				
						6		44 695 547				
4	rs11780978	G/A	0.23	8	145 034 852	1		145 001 361		*PLEC*	Gene body; transcribed	21
						2		145 001 378				
						3		145 001 384				
						4		145 001 406				
						5	cg19405177	145 001 428	3.33 × 10^−17^			
						6		145 001 444				
						7		145 001 464				
						8		145 001 485				
						9	cg14598846	145 008 909	2.72 × 10^−19^			
						10		145 008 918				
						11		145 008 927				
						12		145 008 930				
5	rs3204689	C/G	0.26	15	58 246 802	1	cg12031962	58 353 849	2.0 × 10^−8^	*ALDH1A2*	Intronic; repressed	19,24
						2		58 353 861				
6	rs143384 rs143383	T/C	0.47	20	34 025 756	1	cg14752227	34 000 481	0.010	*GDF5*	Intronic	19
		C/T	0.45		34 025 983	2		34 000 519				
7	rs6516886	T/A	0.41	21	30 393 664	1	cg00065302	30 366 250	0.040	*RWDD2B*	intergenic	21,25
						2	cg05468028	30 391 383	0.006		Intronic; promoter	
						3		30 391 385				
						4	cg18001427	30 391 784	0.010		Intergenic; promoter	
						5	cg20220242	30 392 188	2.40 × 10^−6^			
						6	cg16140273	30 455 616	0.010		Intronic; transcribed	

^a^Putative effector genes at the loci, which have previously shown allelic imbalance in investigations using OA tissues. NEA, non-effect allele; EA, effect allele. Regions were annotated using ROADMAP chondrocyte data (E049).

These data show the tissue-specificity of DNAm in distinct limb tissues during skeletal development. Further, our data show a trend toward active methylation of disease-associated CpGs in chondrocytes during the ageing process.

### OA-associated mQTLs operate during skeletogenesis

To investigate the presence of OA risk mQTLs in the developing human skeleton, DNAm measured in FL and FC tissues at each of the 39 CpGs was stratified by genotype at the association SNV for each respective locus (Supplementary Material, Fig. S2). Significant mQTLs were identified at 14/39 CpGs in FL (*n* = 15–19, *P*_adj_ = 0.047–3.7 × 10^−5^) at the following loci: Locus 1 (4/8 CpGs), Locus 2 (1/3), Locus 3 (2/6), Locus 4 (2/12), Locus 6 (1/2) and Locus 7 (4/6) (Supplementary Material, Fig. S2, Supplementary Material, Table S3). In FC samples, a more comparable tissue to the AC samples in which the mQTLs were originally discovered, significant correlations were identified at 29/39 CpGs (*n* = 54–75, P_adj_ = 0.042–4.4 × 10^−19^; Supplementary Material, Fig. S2, Supplementary Material, Table S3).

Interestingly, at Locus 1 (*COLGALT2*), significant FC mQTLs (*P*_adj_ = 2.00 × 10^−4^–6.60 × 10^−11^) were identified at 7/8 CpGs across an enhancer marked by cg18131582 (CpG7 at the locus), with a mean genotypic effect (GE) of 33.9% across the investigated region (Fig. 1C). No mQTL was detected at CpG1 in any of the investigated tissues, and this CpG was consistently hypermethylated (mean DNAm FL, 93.4%; FC, 84.3%; AC, 83.84%). In AC, mQTLs were identified at only 5/8 CpGs across the enhancer (*P*_adj_ < 0.016) with a mean GE of 13.8% across the region (Fig. 1C). This indicated an OA risk locus with a stronger and physically broader mQTL effect during development. Conversely, at Locus 7 (*RWDD2B*), a stronger mean GE was detected in FL (42.0%) tissues than in either FC (7.5%) or AC (11.2%) (Fig. 1C). This indicated that joint tissues other than cartilage could be the site of the functional mechanism contributing to OA genetic risk at this locus. Furthermore, at Locus 4 (*PLEC*), where we identified a strong contribution of genotype to DNAm in both developmental and aged cartilage (GE, 38.7 and 59.2%, respectively; Fig. 1C), FL samples were hypermethylated (mean DNAm 76.1–98.3%) and no significant mQTLs were observed for 10/12 of the CpGs (*P*_adj_ = 0.82–0.07; GE = 7.6%), pointing toward a cartilage-specific effect.

These data demonstrate the presence of mQTLs associated with OA in the developing human skeleton, indicating that their functional impact could be exerted from the beginning of life, despite the disease manifesting in older age.

### DNAm is co-regulated between CpGs at individual loci

**Figure 2:**
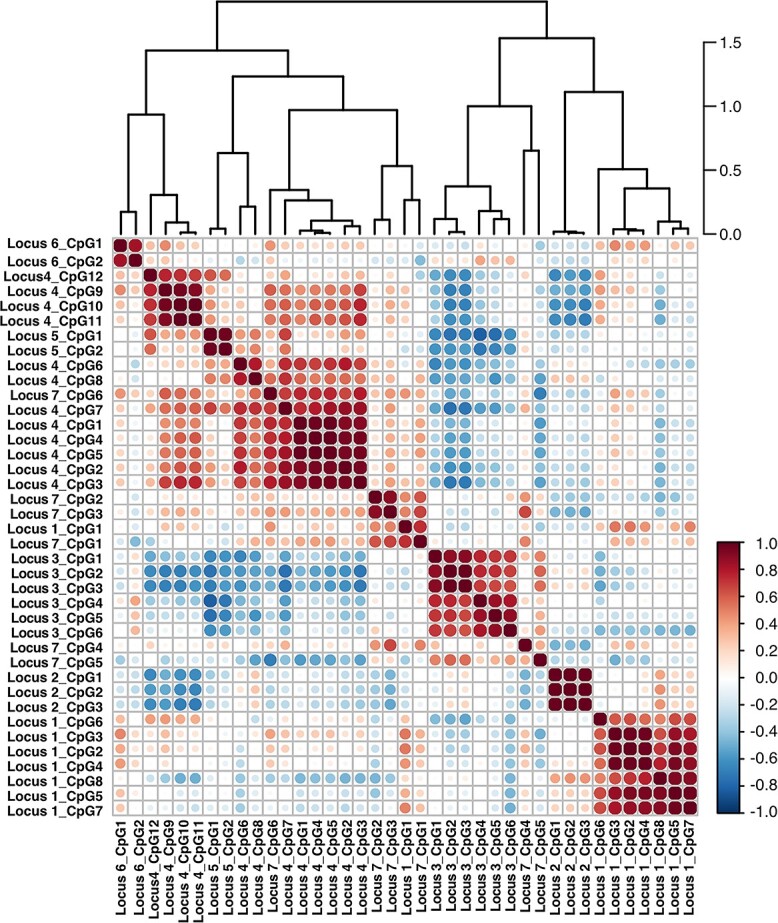
Co-regulation of mQTL CpGs by single SNVs at the loci. Correlation matrix of DNAm values at the 39 CpGs. The color of the circles represents the *r*^2^ correlation (red, strong positive correlation (1.0); white, no correlation (0.0); blue, strong negative correlation (−1.0). The areas of circles show the absolute value of corresponding correlation coefficients and CpGs are ordered using the hierarchical clustering method, the results of which are summarized in the dendrogram above the matrix.

Correlation matrices of DNAm at each of the 39 CpGs were created across all samples (Fig. 2) and in the distinct tissue types (Supplementary Material, Fig. S3). DNAm at CpGs generally clustered by locus, confirming a co-regulation of methylation levels by the association signal (Fig. 2). Notably, at Locus 4 and Locus 7, where multiple mQTL effects have been identified at the individual loci, the CpGs formed distinct clusters. At Locus 4 (harboring *PLEC*), two distinct clusters were identified, with significant negative correlations between the two in FC and AC samples, indicating a single regulatory mechanism with opposing effects upon the two CpG clusters (Supplementary Material, Fig. S3B and C). However, at Locus 7 (harboring *RWDD2B*), where six CpGs were investigated, the three hypomethylated CpGs in physical proximity (CpGs 2–4, located in the *RWDD2B* promoter, Fig. 1A) formed a single cluster (Supplementary Material, Fig. S3), whereas DNAm at the three individual CpGs did not cluster with others at the same locus. This indicated a single genetic signal exerting independent regulatory effects upon multiple CpGs around the *RWDD2B* gene.

Here we have shown that the DNAm levels are co-regulated in cartilage by SNVs at each locus throughout the human life course.

### DNAm correlates with allelic expression of OA risk genes in the developing human skeleton

**Figure 3:**
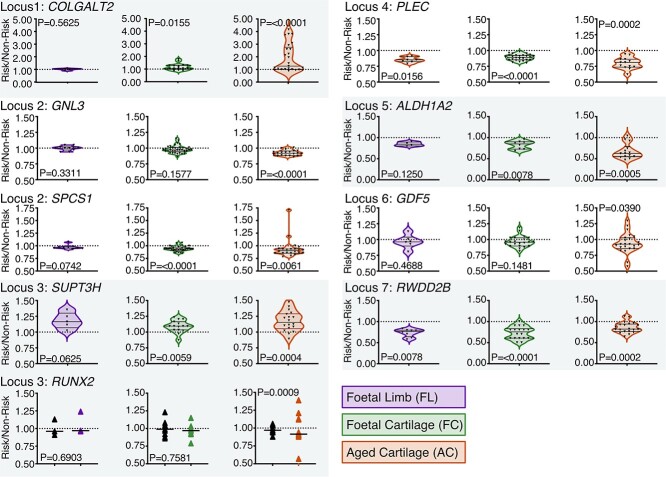
AEI of OA risk genes is present in human developmental limb tissues. Nine genes were investigated across the seven loci. Violin plots represent the allelic ratio in FL (purple), FC (green) and AC (orange) tissues for *COLGALT2* (*n* = FL, 6; FC, 17; AC, 25), *GNL3* (*n* = FL, 11; FC, 34; AC, 20), *SPCS1* (*n* = FL, 9; FC, 32; AC, 22), *SUPT3H* (*n* = FL, 5; FC, 15; AC, 18), *RUNX2* (*n* = FL, 7; FC, 19; AC, 16), *PLEC* (*n* = FL, 7; FC, 27; AC, 13), *ALDH1A2* (*n* = FL, 4; FC, 8; AC, 14), *GDF5* (*n* = FL, 6; FC, 22; AC, 23) and *RWDD2B* (*n* = FL, 8; FC, 18; AC, 20). Individual data points are shown, and horizontal bars represent the median and interquartile range. For *RUNX2*, the transcript variant was in linkage equilibrium (*r*^2^ = 0.0) and so the cDNA allelic ratios were stratified by samples that were homozygous for either the major (A) or minor (G) allele at the association SNV, rs10948172 (black triangles) and those that were heterozygous (colored triangles).

We had previously identified AEI in AC samples at nine genes across the seven investigated loci, identifying them as OA effector genes (21,22,24–28) (Fig. 3 and Supplementary Material, Fig. S4, orange plots). Transcriptome-wide analyses of OA cartilage have also reported AEI at most of these genes (29,30). We first confirmed expression of all nine genes in the developmental tissues (Supplementary Material, Fig. S5) and then investigated the presence of AEI during skeletogenesis. In FL samples, significant AEI (*P* = 0.008–0.016) was identified for *RWDD2B* and *PLEC* (Fig. 3)*.* The relatively small sample size of heterozygous FL tissues at the loci (*n* = 4–11) may have precluded the identification of significant AEI for other genes, namely *SUPT3H* (*P* = 0.063, *n* = 5) and *SPCS1* (*P* = 0.074, *n* = 9) (Supplementary Material, Fig. S4, purple). In FC, significant AEI (*P* < 0.016) was identified at 6/9 genes, including *COLGALT2* (mean allelic ratio = 1.17, *P* = 0.016, *n* = 17), *SPCS1* (0.93, *P* < 0.0001, *n* = 32), *SUPT3H* (1.08, *P* = 0.006, *n* = 15), *PLEC* (0.89, *P* < 0.0001, *n* = 27), *ALDH1A2* (0.81, *P* = 0.008, *n* = 8) and *RWDD2B* (0.73, *P* < 0.0001, *n* = 18). Individual data points for DNA and complementary DNA (cDNA) ratios are displayed in Supplementary Material, Fig. S4. When significant AEI was identified, the direction of the imbalance occurred in the same direction in all investigated tissues.

**Figure 4:**
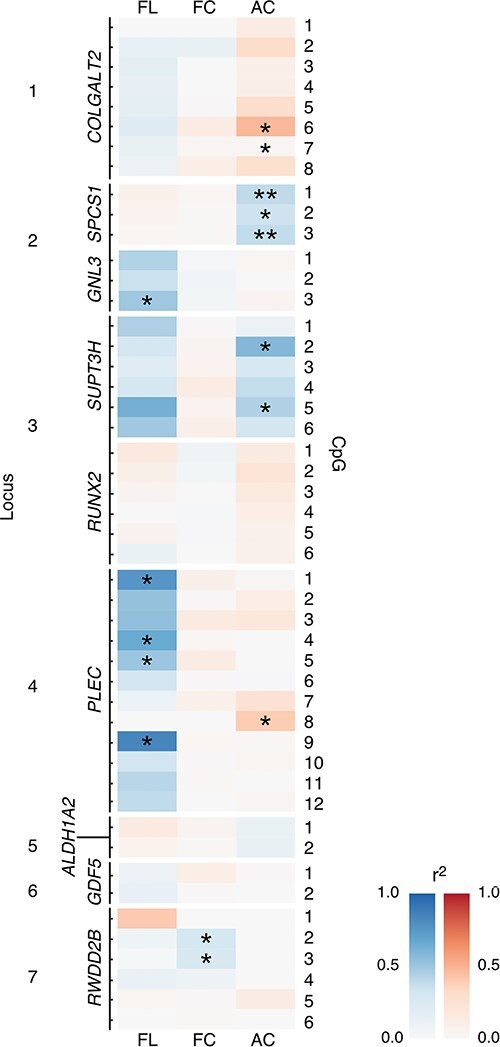
Correlations between DNAm and allelic expression are present in the developing and aged human skeleton. Heatmap showing *r*^2^ values between the Log2 AEI ratio for expression of the nine investigated genes and methylation M-values at each CpG in FL, FC and AC. Negative slopes are colored from white (*r*^2^ = 0.0, no correlation) to blue (*r*^2^ = 1.0, perfect correlation) and positive slopes are colored from white (*r*^2^ = 0.0, no correlation) to red (*r*^2^ = 1.0, perfect correlation). *P*-values were calculated using simple linear regression and corrected for multiple testing using the method of Bonferroni. ^*^^*^P_adj_ < 0.01; ^*^P_adj_ < 0.05.

We next searched for methylation-expression QTLs (meQTLs), whereby DNAm at the CpGs correlated with measured AEI ratios. Methylation M-values were regressed by AEI ratios at each of the nine investigated transcripts (Supplementary Material, Fig. S6). Correlations were identified in all three investigated tissue types and are displayed as a heatmap in Fig. 4. In FL samples, significant meQTLs were detectable at Locus 2 (*GNL3, P* = 0.022) and Locus 4 (*PLEC, P* = 0.028–0.008). In FC, significant meQTLs were detectable at Locus 7 (*RWDD2B, P* = 0.005–0.002). Other loci exhibited correlative trends, namely Locus 3 (*SUPT3H, r*^2^ = 0.29–0.69). However, sample size was often limited for meQTL analysis because of multiple factors including low minor allele frequencies (which limited the number of available heterozygous samples) and low gene expression providing limited mRNA template for amplification (which increased the dropout rate of samples following QC). No correlations were identified between the GE (strength of mQTL) and meQTL *r*^2^ value (impact upon allelic imbalance), indicating that the mQTL effect size is not a predictor of functional impact.

These data show that OA effector genes are differentially expressed at the allelic level in human cartilage and limb tissues from the start of life, and that the mechanisms leading to cartilage loss in older age can be present throughout life. Significant correlations between expression ratios and DNAm indicate a functional role for the observed mQTLs in a spatiotemporal context.

### Identification of open chromatin regions in developmental and AC

**Figure 5:**
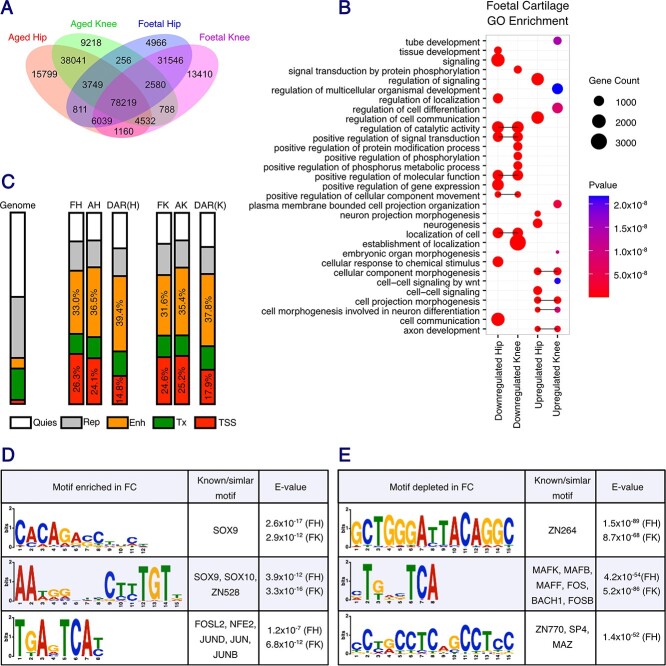
Differential chromatin accessibility between developing and aged hip and knee cartilage. (**A**) Venn diagram summarizing the number of DARs between FH (blue), FK (pink), AH (orange) and AK (green) cartilage. (**B**) GO analysis of the transcripts mapping to the DAR between FC and AC. The size of the circle represents the gene count mapping to the respective GO term and the color represents the *P*-value. Horizontal black bars connect the terms that are upregulated in both FH and FK, or downregulated in both FH and FK. (**C**) Enrichment of peaks mapping to ROADMAP chromatin states in E049 MSC-cultured chondrocytes. The left-hand plot shows the distribution of peaks across the genome. The middle three plots show the peak enrichment in FH and AH samples, along with the enrichment in the differentially accessible hip peaks (DAR(H)). The right-hand plots show the peak enrichment in FK and AK samples, along with the enrichment in the differentially accessible knee peaks (DAR(K)). The percentages of peaks mapping to enhancer (yellow) or promoter (red) regions are annotated. Quies, quiescent; Rep, repressed polycomb; Enh, enhancer; Tx, transcribed region. (**D**) The transcription factor motifs most highly enriched in accessible regions in foetal cartilage. (**E**) The transcription factor motifs most highly enriched in accessible regions in aged cartilage.

We next investigated chromatin accessibility during development and older age. To do so, we conducted an assay for transposase accessible chromatin sequencing (ATAC-seq) in developmental and aged cartilage and primarily analysed this dataset on an epigenome wide scale, before integrating this information with our targeted analysis of OA risk loci. For this investigation, we separately isolated chondrocytes from the human fetal proximal and distal femur (representative of the developing human hip and knee, respectively), along with human AH and AK articular cartilage. We identified 78 219 open chromatin regions that were common across all investigated tissues (Fig. 5A). A total of 49 922 open regions were unique to the fetal samples and 63 058 were unique to the aged samples. Between fetal and aged hip cartilage, 113 887 differentially accessible regions (DAR) were identified [false discovery rate (FDR) < 0.05, Supplementary Material, Table S4]. Similarly, 121 050 DAR were identified between fetal knee (FK) and aged knee (AK) cartilage (Supplementary Material, Table S5).

Gene ontology (GO) analysis of the transcripts mapping to the DAR was performed (Fig. 5B), which showed significant enrichment of cellular component morphogenesis (*P* < 6.0 × 10^−10^), cell projection morphogenesis (*P* < 1.1 × 10^−12^) and cell morphogenesis involved in neuron differentiation (*P* < 8.6 × 10^−9^) in the developing FC, whereas in the AC samples there was a significant enrichment of terms including regulation of catalytic activity (*P* < 4.9 × 10^−13^) and positive regulation of signal transduction (*P* < 5.3 × 10^−12^, Fig. 5B).

Differentially accessible peaks were annotated using ROADMAP chromatin state data generated in mesenchymal stem cell (MSC) differentiated cultured chondrocytes (E049) (Fig. 5C). DAR were significantly overrepresented in gene enhancers yet underrepresented in gene promoters (*P* < 0.0001), confirming that the changes in gene expression between development and older age are predominantly driven by altered enhancer activity. Further analysis of sequence motifs within the DAR identified significant enrichment for transcription factor (TF) binding motifs including SOX9 (*E* < 2.9 × 10^−12^) and JUN (*E* < 1.2 × 10^−7^) within fetal chondrocytes (Fig. 5D, Supplementary Material, Tables S6–S9). In aged chondrocytes, significant motif enrichment was also identified for TFs including ZN264 (*E* < 8.7 × 10^−68^), SP4 and MAZ (*E* = 1.4 × 10^−52^) (Fig. 5E, Supplementary Material, Tables S6–S9). Interestingly, the 5′-TGAGTCA-3′ motif, common to transcription regulators including JUN and FOS was enriched in both up- and downregulated regions in fetal chondrocytes, indicating that the regulation of binding regions for these TFs is integral to chondrocyte homeostasis during both developmental and ageing processes (Fig. 5D and E).

These data identify open (and potentially functional) chromatin regions in developmental and aged cartilage. We identified that the majority of DARs are annotated as enhancers, which are driving the changes in gene expression throughout the life course via altered TF binding.

### Epigenetic risk of OA and chromatin state in human chondrocytes

Intersection of 1005 SNVs in high LD [European ancestry cohorts (EUR *r*^2^ > 0.8] with the investigated association signals with the open chromatin regions prioritized 77 functional variants across the seven investigated loci (Supplementary Material, Table S10). Interestingly, only 32/77 prioritized SNVs intersected with open chromatin regions in all four tissue types, indicating that, if functional, the effects could be exerted at different stages of the life course. In sum, 20 variants intersected with regions uniquely accessible in aged cartilage and 16 were unique to developmental samples (Supplementary Material, Fig. S7, Supplementary Material, Table S10).

**Figure 6:**
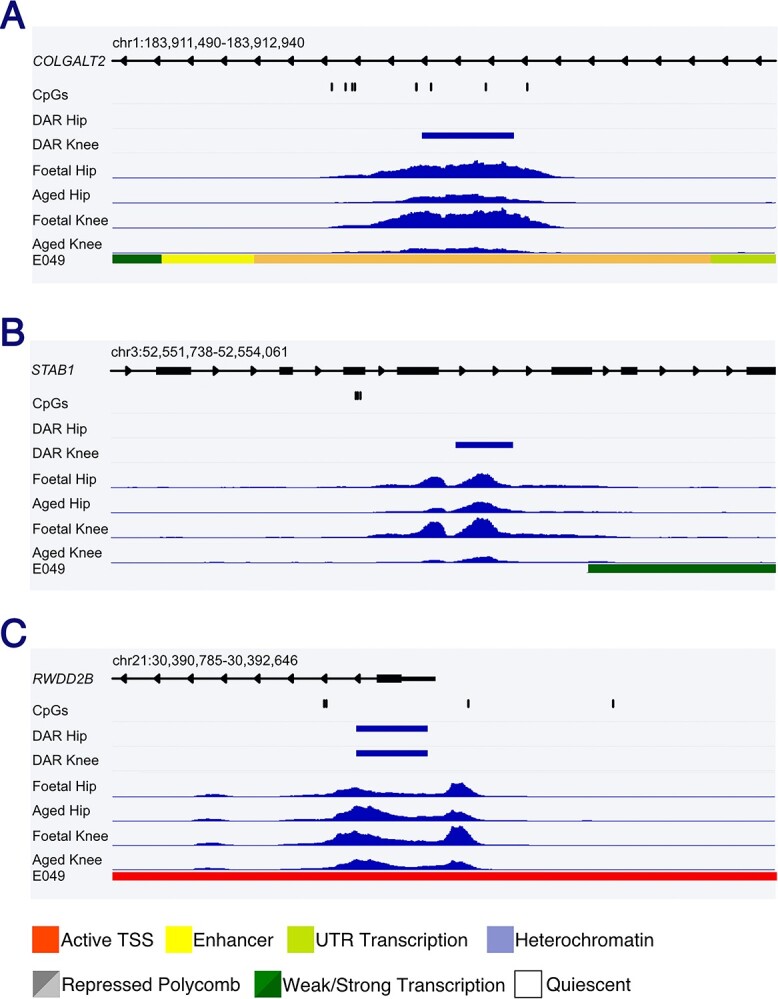
OA-mQTL CpGs fall within DARs in fetal and aged hip and knee cartilage. (**A**) The eight CpGs investigated at Locus 1 fall within an intronic *COLGALT2* enhancer. (**B**) The three intronic CpGs investigated at Locus 2 reside on the edge of an open chromatin region. (**C**) The Locus 7 CpGs that fall within the *RWDD2B* promoter. For (A)–(C), the regions marked as differentially accessible (DAR) in hip and knee cartilage are marked with blue bars. Blue peaks show the average accessibility in each of the four investigated tissue types. The ROADMAP chromatin state in MSC-derived cultured chondrocytes (E049) is summarized at the foot of each diagram.

Finally, we intersected the physical location of the 39 investigated CpGs with open chromatin regions to further indicate likely functional timepoints of the mQTLs at the investigated loci (Table 2). Investigated CpGs fell within open chromatin regions at Locus 1 (*COLGATL2*), 2 (*GNL3/SPCS1*) and 7 (*RWDD2B*). At these genomic positions, peaks were identified across both FC and AC samples; however, all three regions were significantly differentially accessible between fetal and aged chondrocytes (Table 2). At Locus 1 and 2, chromatin was significantly more accessible in FK when compared with AK cartilage (log2 fold change = 0.89, FDR = 0.015; log2 fold change = 0.57, FDR = 0.022, respectively) (Fig. 6A and B). Conversely, at Locus 7, the promoter region of *RWDD2B* was significantly less accessible in both fetal hip (FH, −1.07-fold, FDR = 1.4 × 10^−6^) and FK (−0.51-fold, FDR = 0.012) (Fig. 6C, Supplementary Material, Tables S4 and S5).

**Table 2:** OA-mQTL CpGs fall in open chromatin regions in developmental and AH and AK cartilage

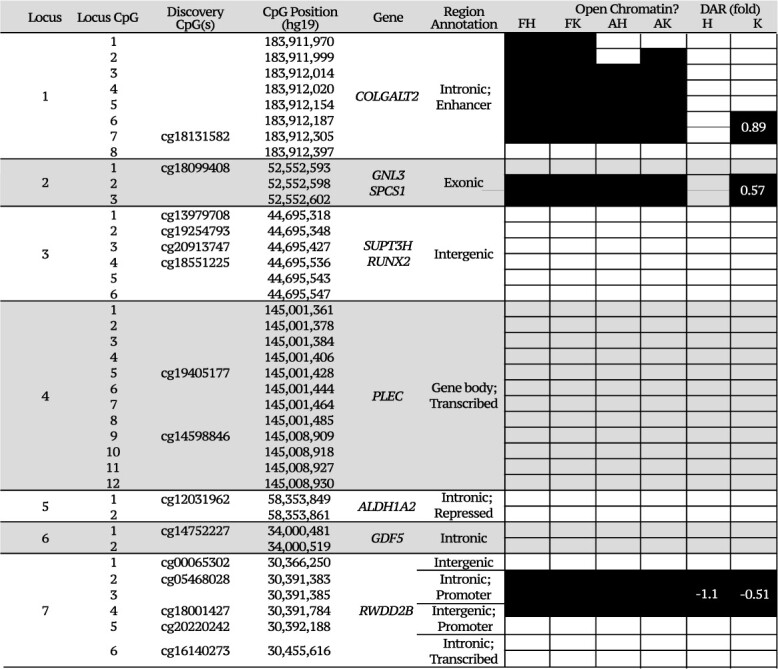

These data have prioritized causal variants falling in open chromatin regions at each of the investigated loci. Finally, we were able to identify in which cartilage tissues our investigated CpGs fell in open regions to provide further evidence for potential timepoints of functional DNAm impact.

## Discussion

DNAm is the most comprehensively investigated human epigenetic mark, and mQTLs have been widely investigated in disease-relevant adult tissues to fine map causal variants and effector genes at genetic risk loci in complex diseases. To date, the investigation of mQTLs during human development has been limited (18,31–33), and studies have predominantly focused upon cerebral tissues and neuropsychiatric disorders. Such analyses have not been previously conducted in the developing human skeleton. In this report, we used a total of 100 human embryonic and fetal skeletal tissue samples to identify differences in DNAm between developmental and aged limbs and investigate the existence of OA genetic risk mechanisms during development.

We investigated 39 CpGs: sites of, or adjacent to, well-characterized OA-mQTLs in AC. We identified significant DMS at 67% of CpGs when comparing developmental and aged cartilage, the majority of which became hypermethylated in aged cartilage. This is noteworthy, as in ageing tissues a trend towards global hypomethylation has been observed, known as the epigenetic drift. The relationship between DNAm and age is complex; however, the consensus is that DNAm increases across tissues in the early years of life, and then gradually declines with age because of a loss in maintenance stringency (34,35). Furthermore, the co-methylation of proximal CpGs has also been observed to decline with older age (36). We identified co-methylation clustering of CpGs at the seven investigated loci. At Locus 4 (*PLEC*) we investigated two distal (7.5 kb) clusters of CpGs (21) at which DNAm was co-regulated, with strong negative correlations between the CpGs, reflecting the 3D architecture of DNA (35). Conversely, at Locus 7, we investigated six mQTL CpGs, only three of which showed a significant co-regulation from the SNV. Importantly, the co-methylation did not appear to be lost with age at any of the loci, suggesting that the epigenetic effects captured by this targeted study are directly relevant to CpGs that are tightly regulated by SNVs and involved in the pathogenesis of tissue-specific diseases.

We identified OA-mQTLs at 28% of investigated CpGs in FL tissues and at 85% in FC. We attribute the relative depletion of mQTLs in the FL samples to both a relatively small sample size (*n* = 19), coupled with mQTL tissue specificity. Whereas this is difficult to disentangle at loci where smaller effect sizes were observed, the absence of FL mQTLs at Locus 4, harbouring *PLEC*, can be attributed to a cartilage-specific effect (CpG 9 GE 71.9% in FC, 6.7% in FL).

One of the most striking observations that we made during this study was the inconsistency in the pattern of observations between the loci, which indicates that the inter-locus mechanisms behind the observed epigenetic changes are distinct. From this, we hypothesize that the functional impacts of these risk loci are exerted at different spatiotemporal points during the human life course. An epigenome-wide study would potentially allow the identification of clusters of loci that show comparable effects and share regulatory timepoints. Yet, in the current targeted study, this is not possible to determine.

Analyses of transcript expression at the nine OA effector genes identified significant AEI at six genes (*P* < 0.016) in FC, confirming the presence of differential transcript expression. This verifies that the functional genetic risk of OA is occurring at the majority of the investigated loci during skeletal development. We investigated meQTLs to determine whether this was being driven by the differential DNAm and identified significant correlations at 15**/**39 CpGs: FL (Locus 2 and 4), FC (Locus 7) and AC (Locus 1, 2, 4). Our inability to widely detect linear correlations between DNAm and target gene expression is unsurprising. The question of DNAm as a cause vs consequence has long been debated, and the answer has been hindered because of the complex involvement of multiple *cis-*regulatory elements (CREs) in the fine-tuning of gene expression. A recent study used single-cell technologies in mouse embryonic stem cells to investigate the co-occurrence of DNAm with chromatin accessibility, and TF occupancy (37). The authors demonstrated that in most enhancers, DNAm neither antagonizes chromatin accessibility nor the binding of TFs. However, they identified a subset of cell-type specific enhancers at which DNAm directly regulates TF binding and target gene expression (37). We postulate that such a subset of methylation-sensitive enhancers would be enriched in targeted mQTL investigations such as ours, where the CREs in which they fall have prior association with disease. This is supported by our recent research using dCas9 epigenome modulators, which have shown a direct causality between DNAm and gene expression at OA loci (22,23,25). The identification of tissue-specific methylation-sensitive enhancers in OA would be bolstered by the expansion of molecular epigenetic investigations to include additional tissues of the articulating joint, rather than solely focusing on cartilage; the inclusion of joint tissues from young adults, although their acquisition in sufficient numbers will be challenging and the adoption of modern single-cell technologies to look for subpopulations of cells driving disease aetiology within the joint.

Finally, we conducted ATAC sequencing and identified open chromatin regions in developmental and aged samples. The identified accessible regions in the FC were enriched for TF binding motifs including SOX9, the master regulatory TF essential for chondrogenesis (38), and FOS/JUN, TFs that can form homo- and heterodimers essential for cartilage development (39–41). Intriguingly, this motif was also enriched in the AC accessible regions. The FOS/JUN TFs have also been shown to play a role in OA pathogenesis (42), and our data further substantiates the notion that stringent regulation of the binding of these TFs to their motifs is integral to cartilage health and function throughout the human life course. We were further able to prioritize causal SNVs across the seven loci, and our data identified three loci at which the investigated CpGs fell into open chromatin regions.

At Locus 1, eight CpGs were investigated across a *COLGALT2* enhancer (22). Generally, levels of DNAm were significantly lower and the GE stronger in the developmental cartilage, consistent with our observation of increased chromatin accessibility during development. Yet, whereas all seven mQTL CpGs were differentially methylated between FC and AC, the difference in mean DNAm at CpG6 was just 4.5%. Additionally, CpG6 was relatively hypomethylated in the AC samples, when compared with the rest of the investigated sites, and this was the site of the highest measured GE at the locus (55.7%). Furthermore, the only significant meQTLs at this locus were identified in AC samples (CpGs 6–7), and whereas AEI for *COLGALT2* was present in both FC and AC samples, the differential expression was greater in AC (mean ratio = 1.95) than in FC (mean ratio = 1.17). We therefore conclude that the biological mechanism underlying OA risk at this locus is established during development; however, the functional impact at this locus is acquired in later life. Notably, the effect size of mQTLs alone does not appear to be a strong predictor of biological function at this locus.

Conversely, Locus 7 appears to be functionally active during fetal development. Here, 3/6 CpGs cluster within the *RWDD2B* promoter and are hypomethylated both in development and older age, consistent with the open chromatin state observed across all tissues. Significant meQTLs were observed in FC samples, indicating that the observed DNAm changes are driving a decrease in *RWDD2B* expression from the beginning of life. *RWDD2B* encodes the RWD domain-containing protein 2B, about which very little is currently known (25). Proteins containing RWD domains are capable of binding to enzymes including ubiquitin ligases (43). This lack of knowledge regarding the biological function of RWDD2B makes it a worthy target of future functional studies in the context of musculoskeletal disease.

*GDF5* (Locus 6) and its regulation have been extensively studied in the context of musculoskeletal development and OA (28,44–48). Identified as a risk gene for OA by the association SNVs rs143383 and rs143384 (49) within the *GDF5* 5′ UTR, early studies into the mechanism behind the regulation revealed AEI for the gene in the human AC and cell models, with the OA risk allele, T, correlating with lower levels of gene expression (28,44,47). More recent, in-depth studies into this locus have utilized murine models, and *in vitro* reporter assays to identify a *Gdf5* enhancer, GROW1, which contains a common derived SNV (rs4911178, G > A) at an otherwise highly conserved position (45). However, to date, the molecular basis of *GDF5* expression has not been investigated in primary human fetal chondrocytes. Surprisingly, we did not identify significant AEI for *GDF5* in FL or FC samples, however, we did observe a significant imbalance in AC samples, consistent with previous studies (28,44). Therefore, we postulate that, in humans, the differential expression of *GDF5* becomes more pronounced throughout the life course, rendering Locus 6 one at which the functional risk of OA is conferred in ageing, with the decreased levels of *GDF5* hindering the ability of chondrocytes to maintain and repair the cartilage tissue in older age (50).

Whilst decades of research have been dedicated to understanding the genetic aetiology of OA, the clinical exploitation of OA genetic discoveries is still out of reach (51). In this report, we undertook a detailed molecular genetic analysis of well-characterized OA risk loci, using primary human musculoskeletal fetal tissues for the first time. Our data indicate that the functional timepoints of OA risk loci can differ, raising the question of whether causal variants could be classified by whether they confer a developmental or age-acquired risk. Our study further confirms that leveraging tissue specific DNAm data can prioritize effector genes and their regulatory elements yet highlights that the effect size of mQTLs alone is not an indicator of function. The translation of genetic discoveries in the OA field requires a deep understanding of the molecular mechanisms by which risk-conferring alleles impact their target genes, and the appropriate timepoint for therapeutic intervention, prior to macroscopic structural damage occurring in the joint (11). This is the first study to identify the presence of mQTLs in human fetal cartilage and limb tissues, and our report demonstrates that the functional genetic risk of OA can be laid down during human skeletogenesis. Strides are being made within the field, with the first recent reports of polygenic risk scores (PRS) for the disease (52,53), yet the clinical utility of such systems is still lacking (54). We would encourage the integration of epigenetic data at the loci, along with clinical and biochemical parameters to further advance these tools for the patient benefit.

## Materials and Methods

### Human fetal sample collection and processing

Human embryonic and fetal tissues were obtained from the MRC and Wellcome Trust funded Human Developmental Biology Resource (HDBR) at Newcastle University (http://www.hdbr.org, project number 200363), with appropriate maternal written consent and approval from the Newcastle and North Tyneside NHS Health Authority Joint Ethics Committee. HDBR is regulated by the UK Human Tissue Authority (HTA; www.hta.gov.uk) and operates in accordance with the relevant HTA Codes of Practice.

The cartilaginous tissue from the ends of developing long bones was specifically isolated and nucleic acids were extracted by the HDBR. Briefly, 20–30 mg of tissue was placed in RLT buffer (Qiagen, 1 053 393) and homogenized with Precelleys lysing kit, CKmix (P000918-LYSko-A) using Precelleys 24 tissue homogenizer (Bertin Technologies). Total RNA and DNA was extracted using an AllPrep DNA/RNA/miRNA Universal Kit (Qiagen, 80 224), according to the manufacturer’s instructions on a QIAGEN QIAcube Automated DNA, RNA isolation machine. The RNA integrity number (RIN) and concentration for each sample was assessed using a 2100 Bioanalyzer (Agilent). Pooled limb tissues were isolated using the same methodology. Summary statistics of the samples are included in Table 3, with full sample details available in Supplementary Material, Figure S1A and B and Supplementary Material, Table S11.

**Table 3:** Summary statistics of the human samples used for molecular genetic analyses

Tissue	Number of samples	Mean developmental age (days)	Age (years)	Sex
Fetal limb	19	56.3 (32–84)	n/a	M, 42.1%; F, 57.9%
Fetal cartilage	75	85.5 (56–119)	n/a	M, 52.0%; F, 48.0%
Adult cartilage	292	n/a	66.9 (25–91)	M, 39.8%; F, 60.2%

Ages are expressed as the mean, with the range in parentheses. M, male; F, female.

### Adult patient sample collection and processing

Human articular cartilage samples were obtained from patients undergoing hip or knee joint replacement surgery because of end-stage OA. The tissue collected was macroscopically intact cartilage, distal from the site of the lesion. Arthroplasty was conducted at the Newcastle upon Tyne NHS Foundation Trust hospitals. The Newcastle and North Tyneside Research Ethics Committee granted ethical approval for the collection, with each donor providing verbal and written informed consent (REC reference number 14/NE/1212). Further details of the patient samples used in this project are provided in Supplementary Material, Table S12 and summarized in Table 3.

RNA was extracted from cartilage by TRIzol-chloroform (Life Technologies) separation, following which the RNA was purified from the aqueous phase using the RNeasy Mini Kit (Qiagen). DNA was extracted from cartilage using the EZNA DNA Isolation kit (Omega Bio-Tek). For genotyping, 50 ng genomic DNA was amplified by PCR as described below. For methylation analysis, 500 ng DNA was deaminated using sodium bisulfite with the EZ DNA Methylation kit (Zymo Research).

### Gene expression analysis

Expression of each of the nine genes of interest was measured across the seven loci in the three investigated tissue types. Complementary DNA (cDNA) was reverse transcribed from total RNA using the Superscript IV standard protocol (Invitrogen) after an initial 15 min treatment with 1 unit of amplification grade DNaseI (Invitrogen). Gene expression was measured by reverse transcription quantitative polymerase chain reaction (RT-qPCR) using pre-designed TaqMan assays (Integrated DNA Technologies, Supplementary Material, Table S13). Gene expression was quantified using TaqMan chemistry, normalized to housekeeping genes *18S, HPRT1* and *GAPDH* and expressed as 2^-Δct^ as described previously (22).

### Pyrosequencing

PyroMark Q24 Advanced (Qiagen) was used to genotype all patient DNA samples as previously described (24). At Locus 4 and 5, restriction fragment length polymorphism assays were used as previously described (21,24). Pyrosequencing was also used to quantify DNAm at the 39 CpGs investigated in this study following bisulfite conversion of DNA (EZ DNA Methylation Kit, Zymo). Each sample was amplified in duplicate. Samples were excluded from the analysis if the replicates differed by > 5%. Assays were designed using PyroMark assay design software 2.0. All primer sequences used for genotyping, methylation and allelic quantification are listed in Supplementary Material, Table S14.

### AEI analysis

AEI analysis was also performed by pyrosequencing as previously described (27). Briefly, samples that were heterozygous for OA association SNVs were genotyped at SNVs falling within one of the nine transcripts of interest. For *COLGALT2, SUPT3H* and *ALDH1A2*, the association SNVs fell within transcribed regions and were used directly for AEI analysis. All other transcript SNVs were in high LD with the association SNV (*r*^2^ > 0.82 in EUR), Supplementary Material, Table S14), except for *RUNX2*, where AEI analysis was performed using a variant in linkage equilibrium as previously described in detail (26). Allelic quantification at the transcript variants was performed in triplicate in DNA and cDNA from each heterozygous sample. The ratio measured in the cDNA was then normalized to the DNA ratio. Samples with triplicate values which differed by > 5% were excluded from analysis.

### Assay for transposase accessible chromatin sequencing (ATAC-seq)

Fresh cartilage tissue from proximal (hip, *n* = 6) and distal (knee, *n* = 6) human fetal femurs at 12 post conception weeks, was dissected and provided by the HDBR. Adult articular cartilage was dissected from hip (*n* = 5) and knee (*n* = 5) joint arthroplasty obtained as described above. For chondrocyte isolation, the cartilage was digested in 1% collagenase solution (Sigma Aldrich) for 3 (fetal) or 16 h (arthroplasty) at 37°C. Cells were washed with 1xPBS, re-suspended in 5%FBS-DMEM and counted. Nuclei were isolated from 50 000 cells by 3 min incubation on ice in lysis buffer (5 M NaCl, 1 M MgCl_2_, 1 M Tris–HCl (pH 7.5), 10% w/v NP-40 (Roche), 10% w/v Tween 20 (Roche) and 1% w/v Digitonin (Promega) (55). Isolated nuclei were re-suspended in a transposase mix (25 μl 2xTD Buffer, 2.5 μl TDE1 enzyme (Nextera Tn5 transposase, Illumina), 0.5 μl 1% w/v Digitonin, 0.5 μl 10% w/v Tween 20, 16.5 μl 1xPBS and 5 μl nuclease-free H_2_O) and incubated for 30 min at 37°C, 1000 rpm. DNA was purified using MinElute PCR Purification kit (Qiagen) according to manufacturer’s instructions and eluted in 10 μl H_2_O. The purified DNA was partially amplified by PCR in a 50 μl reaction using the NEB Next High-Fidelity PCR Master Mix (New England Biolabs, Hitchin, UK) and primer Ad1_noMX in combination with barcoded primers (Supplementary Material, Table S15). A 5 μl aliquot of the reaction was amplified by quantitative PCR (qPCR) and the remaining 45 μl was amplified by PCR for an additional number of cycles, calculated for each sample based on the qPCR readings, to avoid amplification to saturation. The libraries were purified using AMPure XP magnetic beads (Beckman Coulter). The DNA libraries were sequenced by Newcastle University’s Genomics Core Facility on a NextSeq S1 generating paired-end 100 bp reads.

### Bioinformatic and statistical analyses

Pre-processing: The quality of FASTQ files was assessed with FastQC Version 11.8 (56). Adapter sequences were removed using Trimmomatic Version 0.36 (57). Paired-end reads were aligned to GRCh38 using Bowtie2 Version 2.3.4.2 (58). Multi-mapped reads, duplicate reads and reads aligning to the mitochondrial genome were removed using Picard Tools Version 2.2.4 and SAMtools Version 1.9 (59). Peaks were called using MACS2 Version 2.1.1 and ENCODE blacklisted regions were removed from the peak calls using BEDTools Version 2.27.1 (60).

The R package DiffBind (Version 2.16.2) was used to derive consensus peak sets for further analysis. Consensus peak sets consisted of peaks identified in at least 4/6 of the fetal samples, and at least 3/5 of the aged samples. Consensus peak sets were lifted to hg19 using the UCSC liftOver tool and then annotated using the R package ChIPseeker (Version 1.26.2) (61). The Diffbind package was used to determine sites that are differentially accessible between sample groups.

Motif discovery in the differentially accessible sites was performed using MEME-Chip from the MEME Suite (version 5.4.1) (62). Enriched GO terms were determined using the R package GOstats (version 2.56.0) (63). Intersections with CpG sites and ROADMAP chromatin state maps were identified using BEDTools (60).

## Supplementary Material

Supplementary_Figures_ddac251

Supplementary_Tables_S1-S3_ddac251

Supplementary_Table_S4_Chr1-5_ddac251

Supplementary_Table_S4_Chr6-9_ddac251

Supplementary_Table_S4_Chr10-X_ddac251

Supplementary_Table_S5_Chr1-5_ddac251

Supplementary_Table_S5_Chr6-9_ddac251

Supplementary_Table_S5_Chr10-15_ddac251

Supplementary_Table_S5_Chr16-X_ddac251

Supplementary_Tables_S6-S8_ddac251

Supplementary_Tables_S9-S15_ddac251

## Data Availability

All data associated with this study are present in the paper or supplementary materials. The ATAC-sequencing data are available via the Gene Expression Omnibus (GEO, accession number GSE214394).

## References

[1] Prieto-Alhambra, D., Judge, A., Javaid, M.K., Cooper, C., Diez-Perez, A. and Arden, N. (2014) Incidence and risk factors for clinically diagnosed knee, hip and hand osteoarthritis: influences of age, gender and osteoarthritis affecting other joints. Ann. Rheum. Dis., 73, 1659–1664. 23744977 10.1136/annrheumdis-2013-203355 PMC3875433

[2] Hunter, D.J., March, L. and Chew, M. (2020) Osteoarthritis in 2020 and beyond: a Lancet Commission. Lancet, 396, 1711–1712. 33159851 10.1016/S0140-6736(20)32230-3

[3] Kendzerska, T., Jüni, P., King, L.K., Croxford, R., Stanaitis, I. and Hawker, G.A. (2017) The longitudinal relationship between hand, hip and knee osteoarthritis and cardiovascular events: a population-based cohort study. Osteoarthr. Cartil., 25, 1771–1780. 10.1016/j.joca.2017.07.024 28801210

[4] Wang, H., Bai, J., He, B., Hu, X. and Liu, D. (2016) Osteoarthritis and the risk of cardiovascular disease: a meta-analysis of observational studies. Sci. Rep., 6, 39672. 28004796 10.1038/srep39672 PMC5177921

[5] Palazzo, C., Nguyen, C., Lefevre-Colau, M.M., Rannou, F. and Poiraudeau, S. (2016) Risk factors and burden of osteoarthritis. Ann. Phys. Rehabil. Med., 59, 134–138. 26904959 10.1016/j.rehab.2016.01.006

[6] Richard, D., Liu, Z., Cao, J., Kiapour, A.M., Willen, J., Yarlagadda, S., Jagoda, E., Kolachalama, V.B., Sieker, J.T., Chang, G.H. et al. (2020) Evolutionary selection and constraint on human knee chondrocyte regulation impacts osteoarthritis risk. Cell, 181, 362–381. 32220312 10.1016/j.cell.2020.02.057 PMC7179902

[7] Loughlin, J. (2015) Genetic contribution to osteoarthritis development: current state of evidence. Curr. Opin. Rheumatol., 27, 284–288. 25775188 10.1097/BOR.0000000000000171 PMC4423655

[8] Muthuirulan, P., Zhao, D., Young, M., Richard, D., Liu, Z., Emami, A., Portilla, G., Hosseinzadeh, S., Cao, J., Maridas, D. et al. (2021) Joint disease-specificity at the regulatory base-pair level. Nat. Commun., 12, 4161. 34230488 10.1038/s41467-021-24345-9 PMC8260791

[9] Pitsillides, A.A. and Beier, F. (2011) Cartilage biology in osteoarthritis—lessons from developmental biology. Nat. Rev. Rheumatol., 7, 654–663. 21947178 10.1038/nrrheum.2011.129

[10] Qi, Y., Li, B., Wen, Y., Yang, X., Chen, B., He, Z., Zhao, Z., Magdalou, J., Wang, H. and Chen, L. (2021) H3K9ac of TGFβRI in human umbilical cord: a potential biomarker for evaluating cartilage differentiation and susceptibility to osteoarthritis via a two-step strategy. Stem Cell Res. Ther., 12, 163. 33663609 10.1186/s13287-021-02234-8 PMC7934528

[11] Mahmoudian, A., Lohmander, L.S., Mobasheri, A., Englund, M. and Luyten, F.P. (2021) Early-stage symptomatic osteoarthritis of the knee - time for action. Nat. Rev. Rheumatol., 17, 621–632. 34465902 10.1038/s41584-021-00673-4

[12] Swingler, T.E., Wheeler, G., Carmont, V., Elliott, H.R., Barter, M.J., Abu-Elmagd, M., Donell, S.T., Boot-Handford, R.P., Hajihosseini, M.K., Münsterberg, A. et al. (2012) The expression and function of microRNAs in chondrogenesis and osteoarthritis. Arthritis Rheumatol., 64, 1909–1919. 10.1002/art.34314 22143896

[13] Farhang, N., Brunger, J.M., Stover, J.D., Thakore, P.I., Lawrence, B., Guilak, F., Gersbach, C.A., Setton, L.A. and Bowles, R.D. (2017) CRISPR-based epigenome editing of cytokine receptors for the promotion of cell survival and tissue deposition in inflammatory environments. Tissue Eng. Part A, 23, 738–749. 28095751 10.1089/ten.tea.2016.0441 PMC5568019

[14] Aubourg, G., Rice, S.J., Bruce-Wootton, P. and Loughlin, J. (2022) Genetics of osteoarthritis. Osteoarthr. Cartil., 30, 636–649. 10.1016/j.joca.2021.03.002 PMC9067452 33722698

[15] Perzel Mandell, K.A., Eagles, N.J., Wilton, R., Price, A.J., Semick, S.A., Collado-Torres, L., Ulrich, W.S., Tao, R., Han, S., Szalay, A.S. et al. (2021) Genome-wide sequencing-based identification of methylation quantitative trait loci and their role in schizophrenia risk. Nat. Commun., 12, 5251. 34475392 10.1038/s41467-021-25517-3 PMC8413445

[16] Villicaña, S. and Bell, J.T. (2021) Genetic impacts on DNA methylation: research findings and future perspectives. Genome Biol., 22, 127. 33931130 10.1186/s13059-021-02347-6 PMC8086086

[17] Zhang, T., Choi, J., Dilshat, R., Einarsdóttir, B.Ó., Kovacs, M.A., Xu, M., Malasky, M., Chowdhury, S., Jones, K., Bishop, D.T. et al. (2021) Cell-type-specific meQTLs extend melanoma GWAS annotation beyond eQTLs and inform melanocyte gene-regulatory mechanisms. Am. J. Hum. Genet., 108, 1631–1646. 34293285 10.1016/j.ajhg.2021.06.018 PMC8456160

[18] Jaffe, A.E., Gao, Y., Deep-Soboslay, A., Tao, R., Hyde, T.M., Weinberger, D.R. and Kleinman, J.E. (2016) Mapping DNA methylation across development, genotype and schizophrenia in the human frontal cortex. Nat. Neurosci., 19, 40–47. 26619358 10.1038/nn.4181 PMC4783176

[19] Rushton, M.D., Reynard, L.N., Young, D.A., Shepherd, C., Aubourg, G., Gee, F., Darlay, R., Deehan, D., Cordell, H.J. and Loughlin, J. (2015) Methylation quantitative trait locus analysis of osteoarthritis links epigenetics with genetic risk. Hum. Mol. Genet., 24, 7432–7444. 26464490 10.1093/hmg/ddv433 PMC4664171

[20] Rice, S.J., Cheung, K., Reynard, L.N. and Loughlin, J. (2019) Discovery and analysis of methylation quantitative trait loci (mQTLs) mapping to novel osteoarthritis genetic risk signals. Osteoarthr. Cartil., 27, 1545–1556. 10.1016/j.joca.2019.05.017 31173883

[21] Rice, S.J., Tselepi, M., Sorial, A.K., Aubourg, G., Shepherd, C., Almarza, D., Skelton, A.J., Pangou, I., Deehan, D., Reynard, L.N. et al. (2019) Prioritization of PLEC and GRINA as osteoarthritis risk genes through the identification and characterization of novel methylation quantitative trait loci. Arthritis Rheumatol., 71, 1285–1296. 30730609 10.1002/art.40849 PMC6790675

[22] Kehayova, Y.S., Watson, E., Wilkinson, J.M., Loughlin, J. and Rice, S.J. (2021) Genetic and epigenetic interplay within a *COLGALT2* enhancer associated with osteoarthritis. Arthritis Rheumatol., 73, 1856–1865. 33760386 10.1002/art.41738

[23] Rice, S.J., Roberts, J.B., Tselepi, M., Brumwell, A., Falk, J., Steven, C. and Loughlin, J. (2021) Genetic and epigenetic fine-tuning of TGFB1 expression within the human osteoarthritic joint. Arthritis Rheumatol., 73, 1866–1877. 33760378 10.1002/art.41736

[24] Shepherd, C., Zhu, D., Skelton, A.J., Combe, J., Threadgold, H., Zhu, L., Vincent, T.L., Stuart, P., Reynard, L.N. and Loughlin, J. (2018) Functional characterization of the osteoarthritis genetic risk residing at ALDH1A2 identifies rs12915901 as a key target variant. Arthritis Rheumatol., 70, 1577–1587. 29732726 10.1002/art.40545 PMC6175168

[25] Parker, E., Hofer, I., Rice, S.J., Earl, L., Anjum, S.A., Deehan, D.J. and Loughlin, J. (2021) Multi-tissue epigenetic and gene expression analysis combined with epigenome modulation identifies *RWDD2B* as a target of osteoarthritis susceptibility. Arthritis Rheumatol., 73, 100–109. 32755071 10.1002/art.41473

[26] Rice, S.J., Aubourg, G., Sorial, A.K., Almarza, D., Tselepi, M., Deehan, D.J., Reynard, L.N. and Loughlin, J. (2018) Identification of a novel, methylation dependent, *RUNX2* regulatory region associated with osteoarthritis risk. Hum. Mol. Genet., 27, 3464–3474. 30010910 10.1093/hmg/ddy257 PMC6140783

[27] Gee, F., Clubbs, C.F., Raine, E.V.A., Reynard, L.N. and Loughlin, J. (2014) Allelic expression analysis of the osteoarthritis susceptibility locus that maps to chromosome 3p21 reveals cis-acting eQTLs at GNL3 and SPCS1. BMC Med. Genet., 15, 53. 24886551 10.1186/1471-2350-15-53 PMC4101866

[28] Southam, L., Rodriguez-Lopez, J., Wilkins, J.M., Pombo-Suarez, M., Snelling, S., Gomez-Reino, J.J., Chapman, K., Gonzalez, A. and Loughlin, J. (2007) A SNP in the 5'-UTR of GDF5 is associated with osteoarthritis susceptibility in Europeans and with *in vivo* differences in allelic expression in articular cartilage. Hum. Mol. Genet., 16, 2226–2232. 17616513 10.1093/hmg/ddm174

[29] den Hollander, W., Pulyakhina, I., Boer, C., Bomer, N., van der Breggen, R., Arindrarto, W., Coutinho de Almeida, R., Lakenberg, N., Sentner, T., Laros, J.F.J. et al. (2019) Annotating transcriptional effects of genetic variants in disease-relevant tissue: transcriptome-wide allelic imbalance in osteoarthritic cartilage. Arthritis Rheumatol., 71, 561–570. 30298554 10.1002/art.40748 PMC6593438

[30] Coutinho de Almeida, R., Tuerlings, M., Ramos, Y., den Hollander, W., Suchiman, E., Lakenberg, N., Nelissen, R.G.H.H., Mei, H. and Meulenbelt, I. (2022) Allelic expression imbalance in articular cartilage and subchondral bone refined genome-wide association signals in osteoarthritis. Rheumatology, (in press). 10.1093/rheumatology/keac498 PMC10070069 36040165

[31] Hoffmann, A., Ziller, M. and Spengler, D. (2016) The future is the past: methylation QTLs in schizophrenia. Genes (Basel), 7, 104. 27886132 10.3390/genes7120104 PMC5192480

[32] Andrews, S.V., Ellis, S.E., Bakulski, K.M., Sheppard, B., Croen, L.A., Hertz-Picciotto, I., Newschaffer, C.J., Feinberg, A.P., Arking, D.E., Ladd-Acosta, C. et al. (2017) Cross-tissue integration of genetic and epigenetic data offers insight into autism spectrum disorder. Nat. Commun., 8, 1011. 29066808 10.1038/s41467-017-00868-y PMC5654961

[33] Bonder, M.J., Kasela, S., Kals, M., Tamm, R., Lokk, K., Barragan, I., Buurman, W.A., Deelen, P., Greve, J.W., Ivanov, M. et al. (2014) Genetic and epigenetic regulation of gene expression in foetal and adult human livers. BMC Genomics, 15, 860. 25282492 10.1186/1471-2164-15-860 PMC4287518

[34] Jones, M.J., Goodman, S.J. and Kobor, M.S. (2015) DNA methylation and healthy human aging. Aging Cell, 14, 924–932. 25913071 10.1111/acel.12349 PMC4693469

[35] Seale, K., Horvath, S., Teschendorff, A., Eynon, N. and Voisin, S. (2022) Making sense of the ageing methylome. Nat. Rev. Genet., 23, 585–605. 35501397 10.1038/s41576-022-00477-6

[36] Heyn, H., Li, N., Ferreira, H.J., Moran, S., Pisano, D.G., Gomez, A., Diez, J., Sanchez-Mut, J.V., Setien, F., Carmona, F.J. et al. (2012) Distinct DNA methylomes of newborns and centenarians. Proc. Natl. Acad. Sci. USA, 109, 10522–10527. 22689993 10.1073/pnas.1120658109 PMC3387108

[37] Kreibich, E., Kleinendorst, R., Barzaghi, G., Kaspar, S. and Krebs, A.R. (2022) Single molecule multi-omics reveals context-dependent regulation of enhancers by DNA methylation. *bioRxiv* 2022.05.19.492653. 10.1101/2022.05.19.492653. 36758546

[38] Lefebvre, V., Angelozzi, M. and Haseeb, A. (2019) SOX9 in cartilage development and disease. Curr. Opin. Cell Biol., 61, 39–47. 31382142 10.1016/j.ceb.2019.07.008 PMC6956855

[39] Papachristou, D., Pirttiniemi, P., Kantomaa, T., Agnantis, N. and Basdra, E.K. (2006) Fos- and Jun-related transcription factors are involved in the signal transduction pathway of mechanical loading in condylar chondrocytes. Eur. J. Orthod., 28, 20–26. 16373449 10.1093/ejo/cji101

[40] Mechta-Grigoriou, F., Gerald, D. and Yaniv, M. (2001) The mammalian Jun proteins: redundancy and specificity. Oncogene, 20, 2378–2389. 11402334 10.1038/sj.onc.1204381

[41] Karreth, F., Hoebertz, A., Scheuch, H., Eferl, R. and Wagner, E.F. (2004) The AP1 transcription factor Fra2 is required for efficient cartilage development. Development, 131, 5717–5725. 15509771 10.1242/dev.01414

[42] Neefjes, M., van Caam, A.P.M. and van der Kraan, P.M. (2020) Transcription factors in cartilage homeostasis and osteoarthritis. Biology (Basel), 9, 290. 32937960 10.3390/biology9090290 PMC7563835

[43] Alontaga, A.Y., Ambaye, N.D., Li, Y.J., Vega, R., Chen, C.H., Bzymek, K.P., Williams, J.C., Hu, W. and Chen, Y. (2015) RWD domain as an E2 (Ubc9)-interaction module. J. Biol. Chem., 290, 16550–16559. 25918163 10.1074/jbc.M115.644047 PMC4505409

[44] Egli, R.J., Southam, L., Wilkins, J.M., Lorenzen, I., Pombo-Suarez, M., Gonzalez, A., Carr, A., Chapman, K. and Loughlin, J. (2009) Functional analysis of the osteoarthritis susceptibility-associated *GDF5* regulatory polymorphism. Arthritis Rheumatol., 60, 2055–2064. 10.1002/art.24616 PMC6860363 19565498

[45] Capellini, T.D., Chen, H., Cao, J., Doxey, A.C., Kiapour, A.M., Schoor, M. and Kingsley, D.M. (2017) Ancient selection for derived alleles at a GDF5 enhancer influencing human growth and osteoarthritis risk. Nat. Genet., 49, 1202–1210. 28671685 10.1038/ng.3911 PMC6556117

[46] Chen, H., Capellini, T.D., Schoor, M., Mortlock, D.P., Reddi, A.H. and Kingsley, D.M. (2016) Heads, shoulders, elbows, knees, and toes: modular Gdf5 enhancers control different joints in the vertebrate skeleton. PLoS Genet., 12, e1006454. 27902701 10.1371/journal.pgen.1006454 PMC5130176

[47] Syddall, C.M., Reynard, L.N., Young, D.A. and Loughlin, J. (2013) The identification of trans-acting factors that regulate the expression of GDF5 via the osteoarthritis susceptibility SNP rs143383. PLoS Genet., 9, e1003557. 23825960 10.1371/journal.pgen.1003557 PMC3694828

[48] Reynard, L.N., Bui, C., Syddall, C.M. and Loughlin, J. (2014) CpG methylation regulates allelic expression of GDF5 by modulating binding of SP1 and SP3 repressor proteins to the osteoarthritis susceptibility SNP rs143383. Hum. Genet., 133, 1059–1073. 24861163 10.1007/s00439-014-1447-z PMC4099533

[49] Miyamoto, Y., Mabuchi, A., Shi, D., Kubo, T., Takatori, Y., Saito, S., Fujioka, M., Sudo, A., Uchida, A., Yamamoto, S. et al. (2007) A functional polymorphism in the 5' UTR of GDF5 is associated with susceptibility to osteoarthritis. Nat. Genet., 39, 529–533. 17384641 10.1038/2005

[50] Kania, K., Colella, F., Riemen, A., Wang, H., Howard, K.A., Aigner, T., Dell'Accio, F., Capellini, T.D., Roelofs, A.J. and De Bari, C. (2020) Regulation of Gdf5 expression in joint remodelling, repair and osteoarthritis. Sci. Rep., 10, 157. 31932746 10.1038/s41598-019-57011-8 PMC6957535

[51] Loughlin, J. (2022) Translating osteoarthritis genetics research: challenging times ahead. Trends Mol. Med., 28, 176–182. 35033441 10.1016/j.molmed.2021.12.007

[52] Lacaze, P., Wang, Y., Polekhina, G., Bakshi, A., Riaz, M., Owen, A., Franks, A., Abidi, J., Tiller, J., McNeil, J. et al. (2022) Genomic risk score for advanced osteoarthritis in older adults. Arthritis Rheumatol., 74, 1480–1487. 35506208 10.1002/art.42156 PMC9427681

[53] Sedaghati-Khayat, B., Boer, C.G., Runhaar, J., Bierma-Zeinstra, S., Broer, L., Ikram, M.A., Zeggini, E., Uitterlinden, A.G., van Rooij, J. and van Meurs, J. (2022) Risk assessment for hip and knee osteoarthritis using polygenic risk scores. Arthritis Rheumatol., 74, 1488–1496. 35644035 10.1002/art.42246 PMC9541521

[54] Yau, M.S. and Loughlin, J. (2022) Towards precision medicine - is genetic risk prediction ready for prime time in osteoarthritis? Arthritis Rheumatol., 74, 1477–1479. 35522793 10.1002/art.42155 PMC9427682

[55] Buenrostro, J.D., Wu, B., Chang, H.Y. and Greenleaf, W.J. (2015) ATAC-seq: a method for assaying chromatin accessibility genome-wide. Curr. Protoc. Mol. Biol., 109, 21.29.1–21.29.9. 10.1002/0471142727.mb2129s109 PMC4374986 25559105

[56] Andrews, S . (2010) FastQC: a quality control tool for high throughput sequence data, available online at http://www.bioinformatics.babraham.ac.uk/projects/fastqc/.

[57] Langmead, B., Trapnell, C., Pop, M. and Salzberg, S.L. (2009) Ultrafast and memory-efficient alignment of short DNA sequences to the human genome. Genome Biol., 10, R25. 19261174 10.1186/gb-2009-10-3-r25 PMC2690996

[58] Danecek, P., Bonfield, J.K., Liddle, J., Marshall, J., Ohan, V., Pollard, M.O., Whitwham, A., Keane, T., McCarthy, S.A., Davies, R.M. et al. (2021) Twelve years of SAMtools and BCFtools. Gigascience, 10, giab008. 33590861 10.1093/gigascience/giab008 PMC7931819

[59] Zhang, Y., Liu, T., Meyer, C.A., Eeckhoute, J., Johnson, D.S., Bernstein, B.E., Nusbaum, C., Myers, R.M., Brown, M., Li, W. et al. (2008) Model-based analysis of ChIP-Seq (MACS). Genome Biol., 9, R137. 18798982 10.1186/gb-2008-9-9-r137 PMC2592715

[60] Quinlan, A.R. and Hall, I.M. (2010) BEDTools: a flexible suite of utilities for comparing genomic features. Bioinformatics, 26, 841–842. 20110278 10.1093/bioinformatics/btq033 PMC2832824

[61] Yu, G., Wang, L.G. and He, Q.Y. (2015) ChIPseeker: an R/Bioconductor package for ChIP peak annotation, comparison and visualization. Bioinformatics, 31, 2382–2383. 25765347 10.1093/bioinformatics/btv145

[62] Machanick, P. and Bailey, T.L. (2011) MEME-ChIP: motif analysis of large DNA datasets. Bioinformatics, 27, 1696–1697. 21486936 10.1093/bioinformatics/btr189 PMC3106185

[63] Falcon, S. and Gentleman, R. (2007) Using GOstats to test gene lists for GO term association. Bioinformatics, 23, 257–258. 17098774 10.1093/bioinformatics/btl567

