# Supplementary material for: Genetic risk of osteoarthritis operates during human skeletogenesis
Source: Hum Mol Genet. 2022 Oct 9;32(13):2124–38. doi: 10.1093/hmg/ddac251 (PMC10281754; doi:10.1093/hmg/ddac251)
Supplement: Supplementary_Figures_ddac251 [file supplementary_figures_ddac251.pdf]

Genetic risk of osteoarthritis operates during human skeletogenesis

Supplementary figures

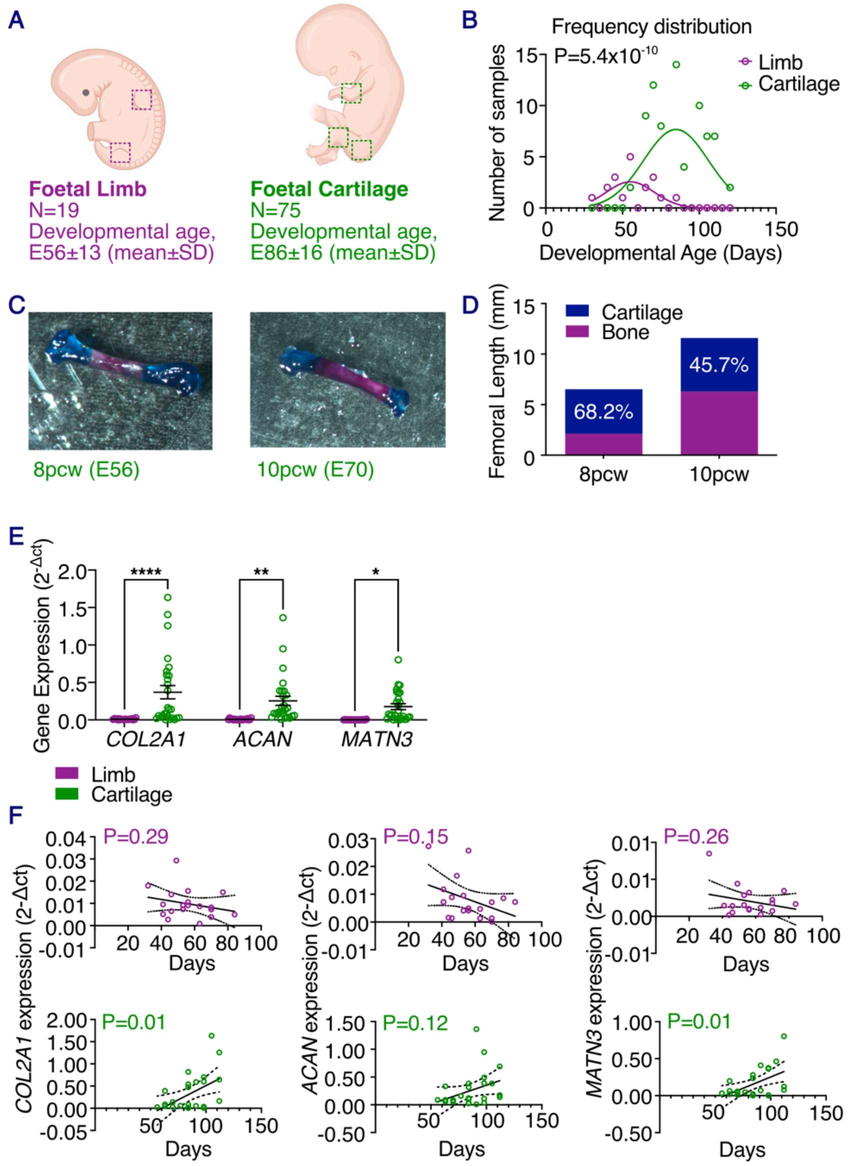

Supplementary Figure 1. Characterization of the foetal tissues utilized in this study. **(A)** Schematic diagram of the tissue types used in this study. **(B)** Histogram showing the distribution of the foetal limb (FL) and foetal cartilage (FC) sample ages used for analysis. Significance was calculated between the two sample types using a t-test. **(C)** Alizarin red (bone) and alcian blue (cartilage) staining of the human foetal femur at 8 post conception weeks (pcw) (left-hand panel) and 10 pcw (right-hand panel). **(D)** Quantification of the images shown in (C). **(E)** Gene expression analysis of cartilage markers *COL2A1, ACAN,* and *MATN3*. Purple, FL; green, FC. Significance was calculated using a 2-way analysis of variance (ANOVA) test. ****, P<0.0001; **, P<0.01; *, P<0.05. **(F)** Plots of simple linear correlation between the expression of the genes in (E) in FL (purple) and FC (green) samples and developmental age. The regression slopes (solid line) and 95% CI (dashed line) are shown in black.

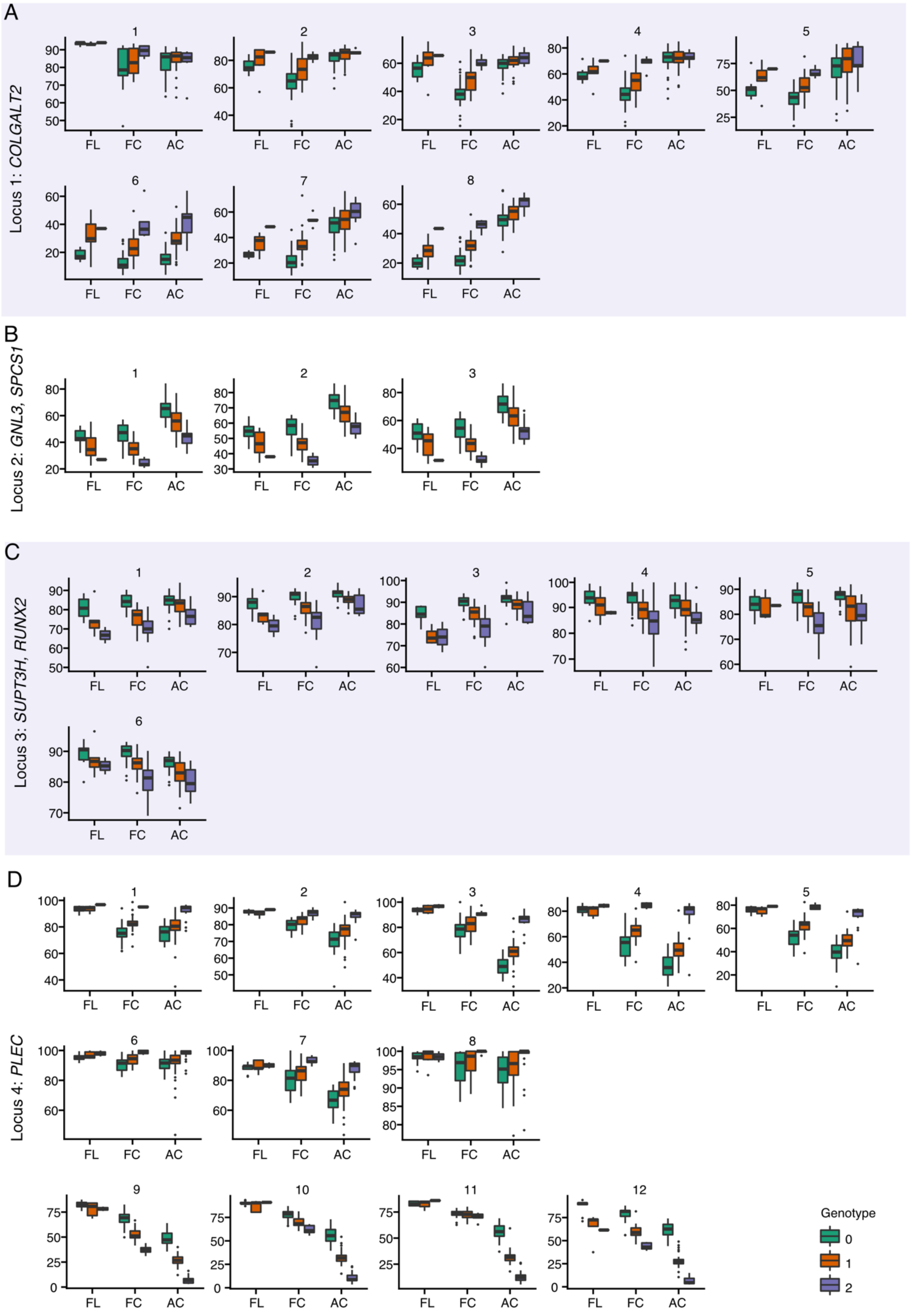

A
Locus 1: COLGALT2
B
Locus 2: GNL3, SPCS1
C
Locus 3: SUPT3H, RUNX2
D
Locus 4: PLEC
FL
FC
AC
Genotype
0
1
2

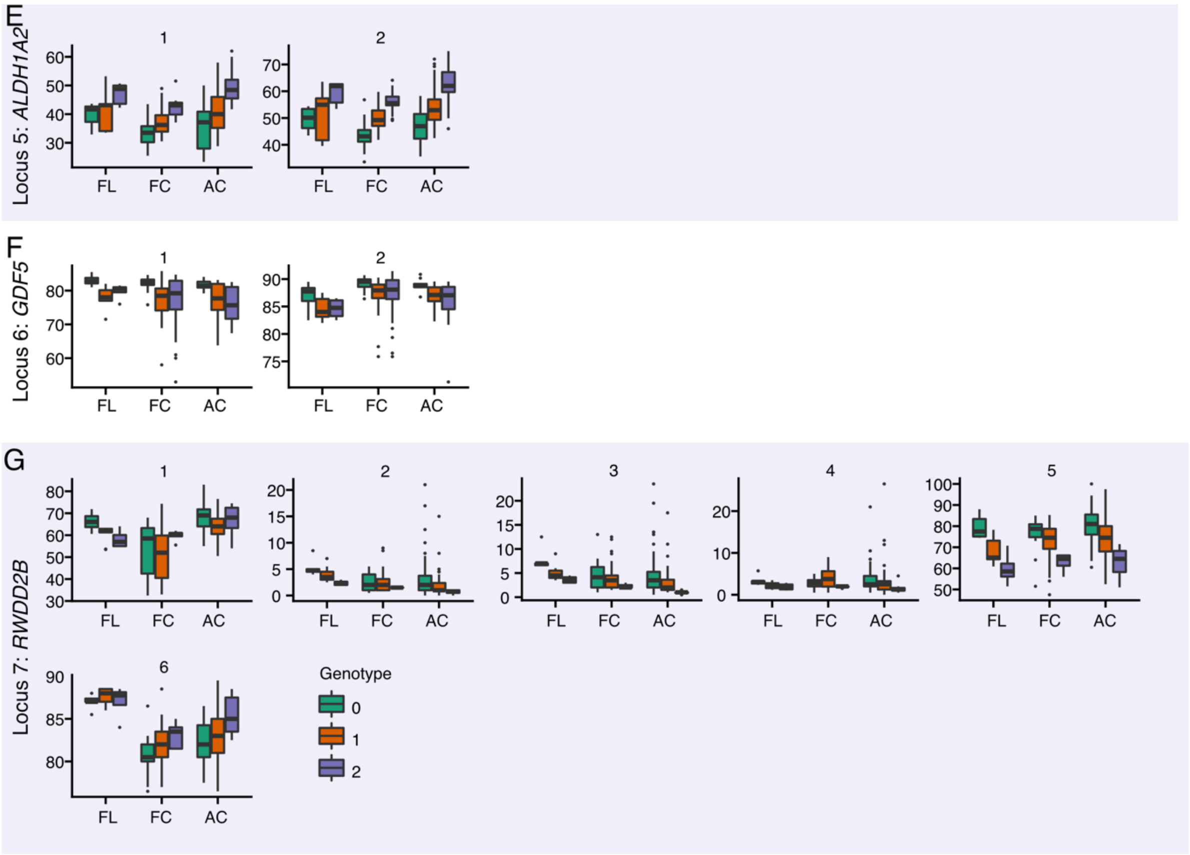

Supplementary Figure 2. mQTL plots of the 39 investigated CpGs. Box and whisker plots of DNAm at the 39 CpGs, stratified by SNV genotype at the appropriate association signal in foetal limb (FL), foetal cartilage (FC) and aged cartilage (AC samples). Green, major allele homozygote; orange, heterozygote; purple, minor allele homozygote. Full details of sample number and statistics are summarized in Supplementary Table 8. **(A)** Locus 1 (*COLGALT2*); **(B)** Locus 2 (*GNL3, SPCS1*); **(C)** Locus 3 (*SUPT3H, RUNX2*); **(D)** Locus 4 (*PLEC*); **(E)** Locus 5 (*ALDH1A2*); **(F)** Locus 6 (*GDF5*); **(G)** Locus 7 (*RWDD2B*).

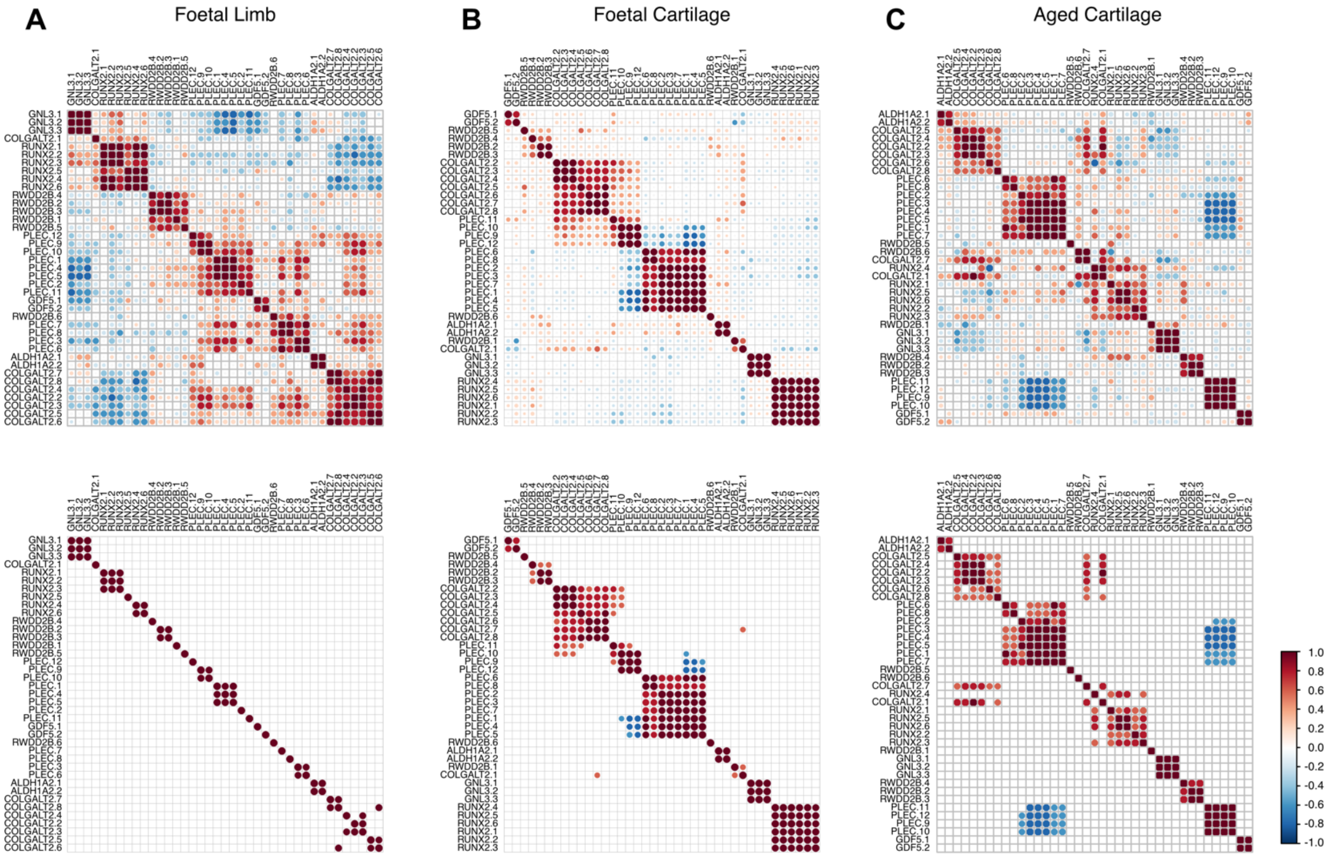

Supplementary Figure 3. Co-regulation of mQTL CpGs by single SNVs in distinct tissues. Correlation matrices of DNAm values at the 39 CpGs. **(A)** Correlations in foetal limb; **(B)** foetal cartilage; **(C)** aged cartilage. Upper panels show all correlations, whilst lower panels show significant correlations calculated in the R package CorrPlot with a significance threshold of P<0.05. The colour of the circles represents the r^2 correlation (red, strong positive correlation (1.0); white, no correlation (0.0); blue, strong negative correlation (-1.0). The areas of circles show the absolute value of corresponding correlation coefficients and CpGs are ordered using the hierarchical clustering method.

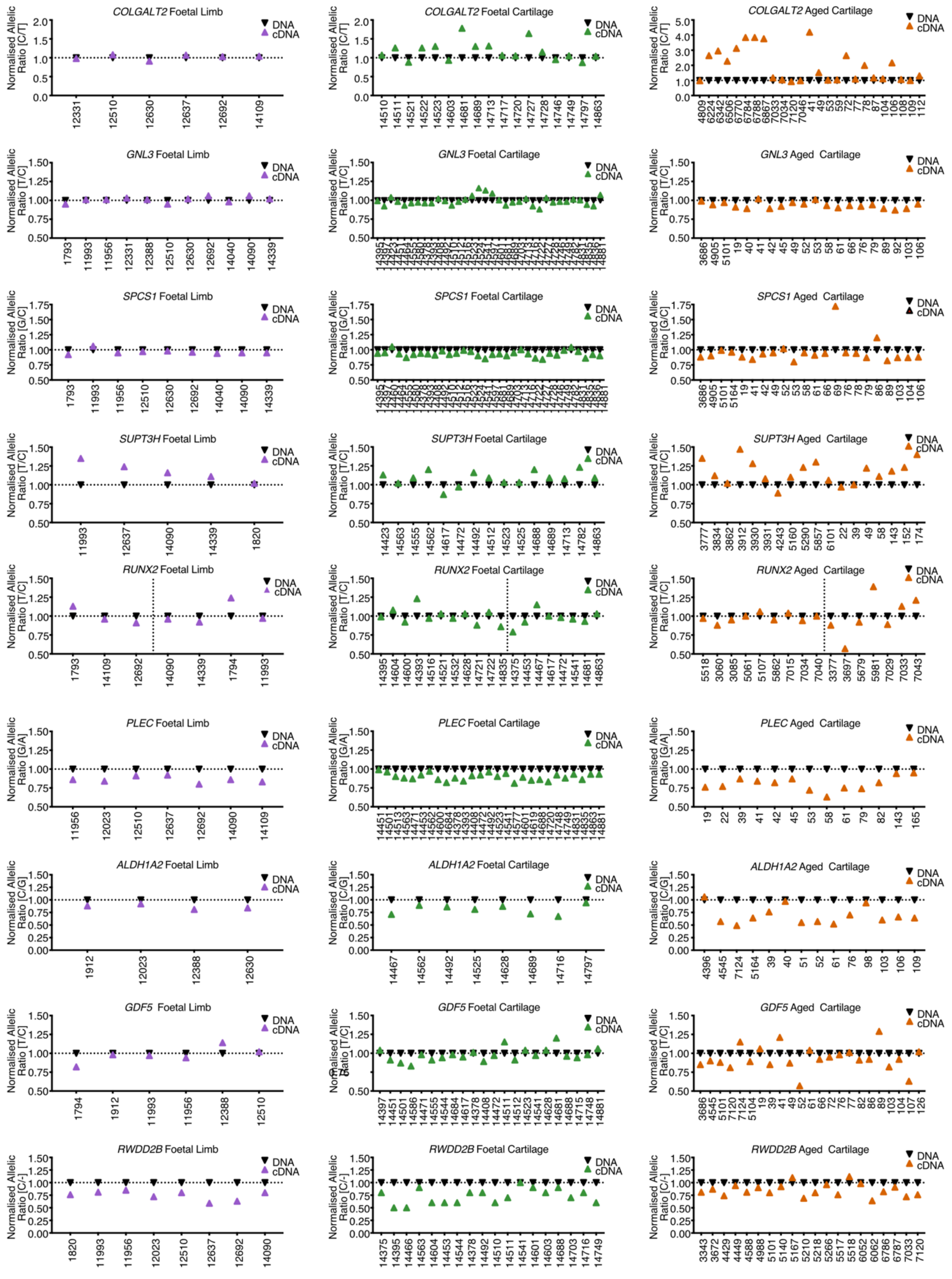

Supplementary Figure 4. Allelic expression imbalance of OA risk genes in human developmental limb tissues. Nine genes were investigated across the seven loci. Individual data points represent the mean allelic ratio in DNA (black) and cDNA in foetal limb (purple), foetal cartilage (green) and aged cartilage (orange) tissues for *COLGALT2* (n= FL, 6; FC, 17; AC, 25), *GNL3* (n= FL, 11; FC, 34; AC, 20), *SPCS1* (n= FL, 9; FC, 32; AC, 22), *SUPT3H* (n= FL,

5; FC, 15; AC, 18), *RUNX2* (n= FL, 7; FC, 19; AC, 16), *PLEC* (n= FL, 7; FC, 27; AC, 13), *ALDH1A2* (n= FL, 4; FC, 8; AC, 14), *GDF5* (n= FL, 6; FC, 22; AC, 23), and *RWDD2B* (n= FL, 8; FC, 18; AC, 20). For *RUNX2*, the samples on the left-hand side of the vertical dotted line represent those that were homozygous for either the major (A) or minor (G) allele at the association SNV, rs10948172 and those on the right-hand side were heterozygous.

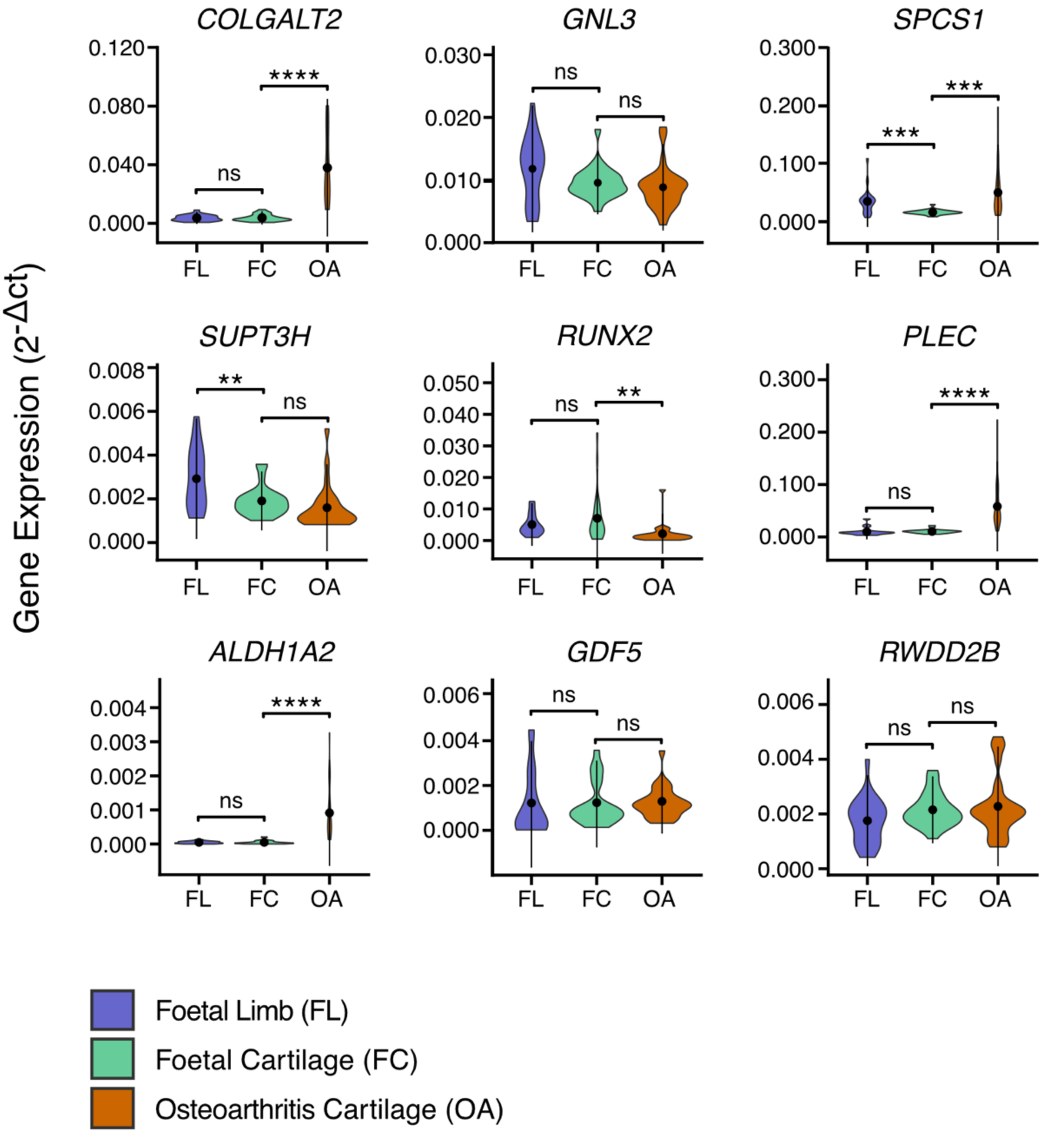

Supplementary Figure 5. Gene expression of the nine OA risk genes in foetal and aged samples. Violin plots showing expression of the nine genes quantified using RT-qPCR normalized to the housekeeping genes *GAPDH, HPRT1,* and *18S* and expressed using the method of $2^{-\Delta ct}$. Expression was quantified in foetal limb (FL, purple, n=19-21), foetal cartilage (FC, green, n=18-25), and aged cartilage (AC, orange, n=24-26) samples. Black circles represent the mean, vertical lines represent the standard deviation. ****, $P_{adj}<0.0001$; ***, $P_{adj}<0.001$; **, $P_{adj}<0.01$; *, $P_{adj}<0.05$; ns, not significant ($P_{adj}>0.05$). Statistical significance was calculated using multiple t-tests with Benjamini-Hochberg correction.

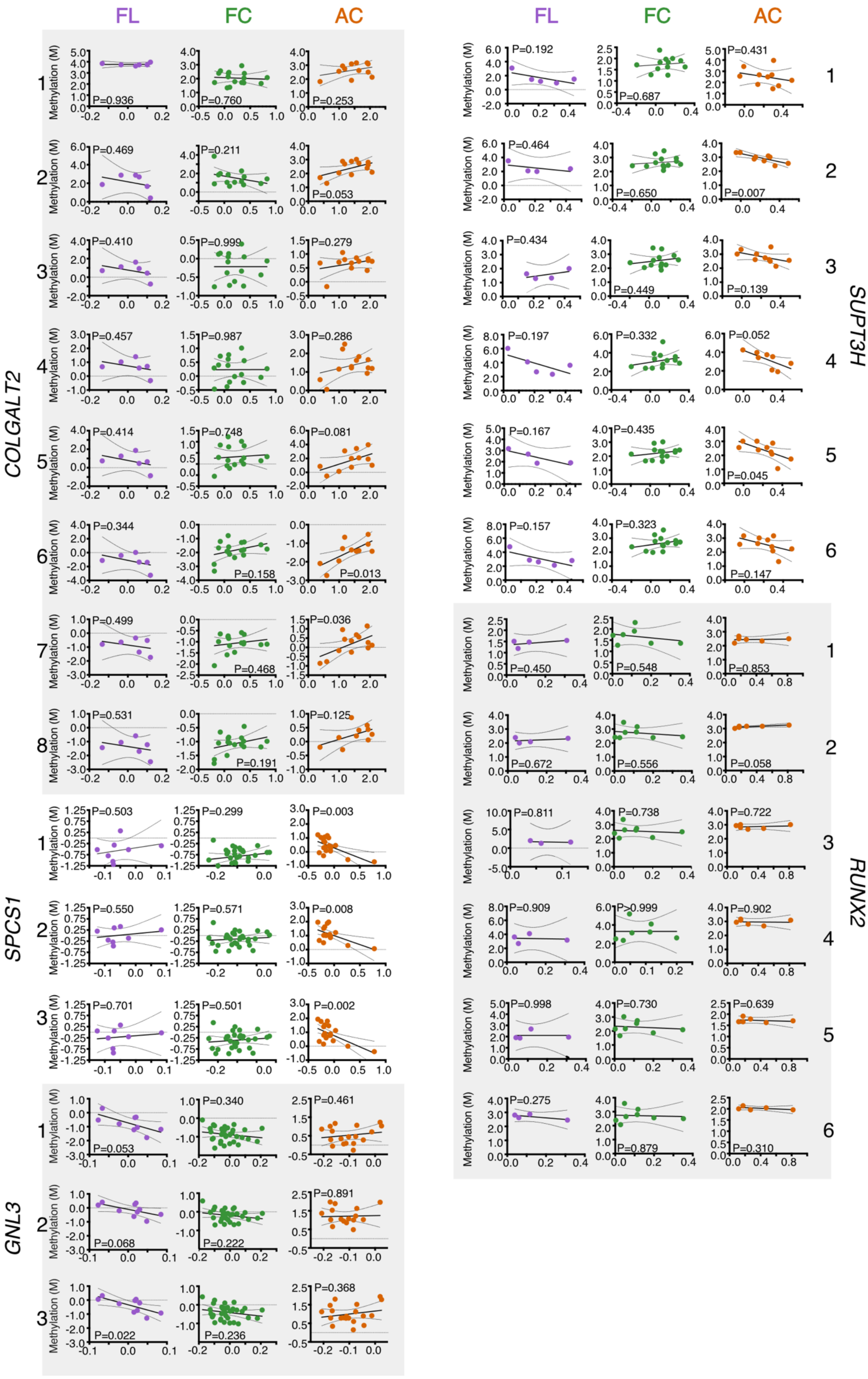
FL
FC
AC
Methylation (M)
COLGALT2
1
P=0.936
P=0.760
P=0.253
2
P=0.469
P=0.211
P=0.053
3
P=0.410
P=0.999
P=0.279
4
P=0.457
P=0.987
P=0.286
5
P=0.414
P=0.748
P=0.081
6
P=0.344
P=0.158
P=0.013
7
P=0.499
P=0.468
P=0.036
8
P=0.531
P=0.191
P=0.125
SPCS1
1
P=0.503
P=0.299
P=0.003
2
P=0.550
P=0.571
P=0.008
3
P=0.701
P=0.501
P=0.002
GNL3
1
P=0.053
P=0.340
P=0.461
2
P=0.068
P=0.222
P=0.891
3
P=0.022
P=0.236
P=0.368
SUPT3H
1
P=0.192
P=0.687
P=0.431
2
P=0.464
P=0.650
P=0.007
3
P=0.434
P=0.449
P=0.139
4
P=0.197
P=0.332
P=0.052
5
P=0.167
P=0.435
P=0.045
6
P=0.157
P=0.323
P=0.147
RUNX2
1
P=0.450
P=0.548
P=0.853
2
P=0.672
P=0.556
P=0.058
3
P=0.811
P=0.738
P=0.722
4
P=0.909
P>0.999
P=0.902
5
P=0.998
P=0.730
P=0.639
6
P=0.275
P=0.879
P=0.310

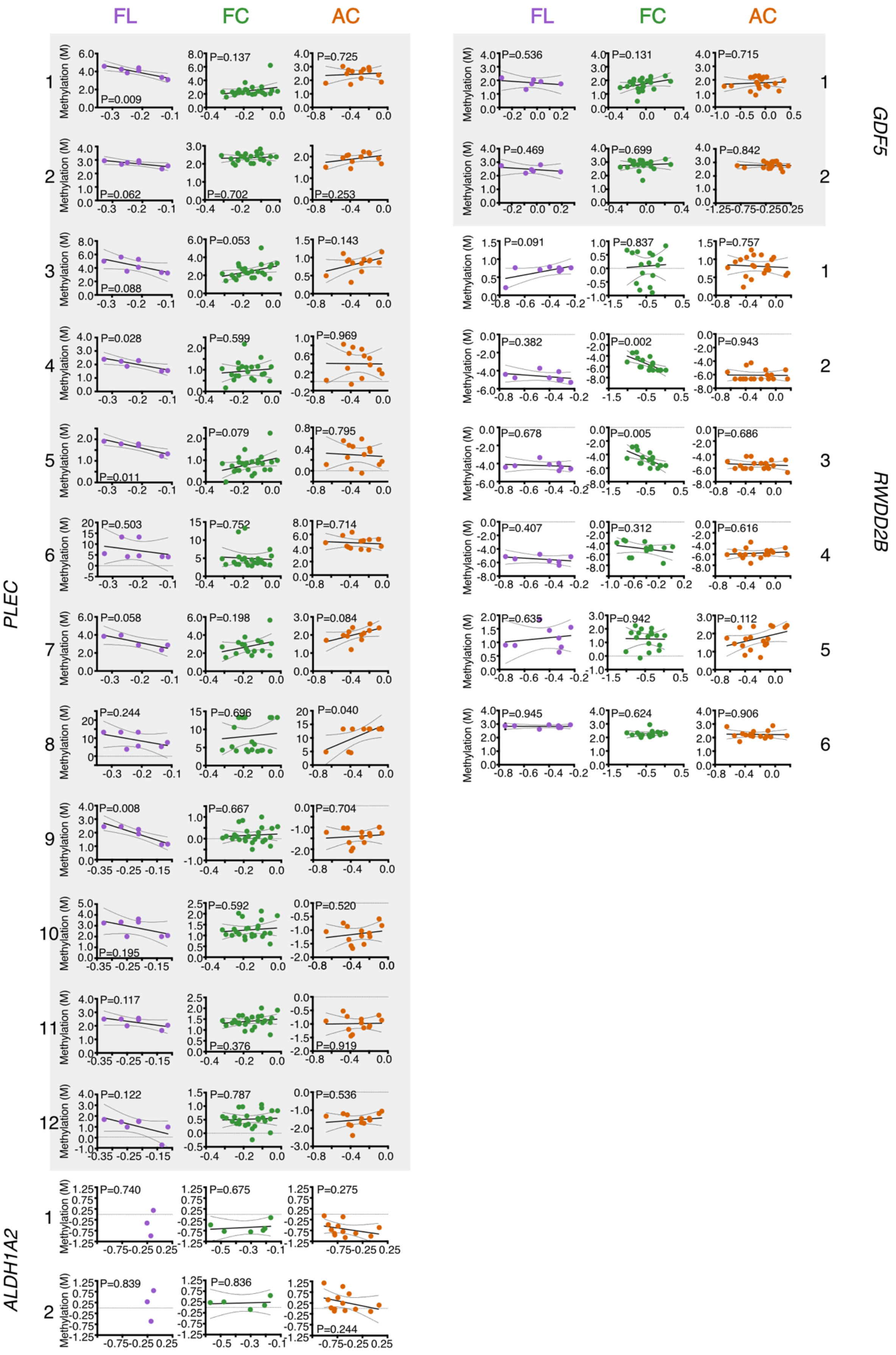

Supplementary Figure 6. Methylation-expression correlations in human tissues from the

developing and aged limb. Graphs show the slope (solid black line) and 95% confidence intervals (dashed line) of the correlations between the Log2 AEI ratio (x-axis), and the methylation M values (y-axis) at each CpG in foetal limb (FL, purple), foetal cartilage (FC, green), and aged cartilage (AC, orange). The horizontal dotted line is at zero. Correlations were calculated using simple linear regression and P-values were corrected for multiple testing using the method of Bonferroni.

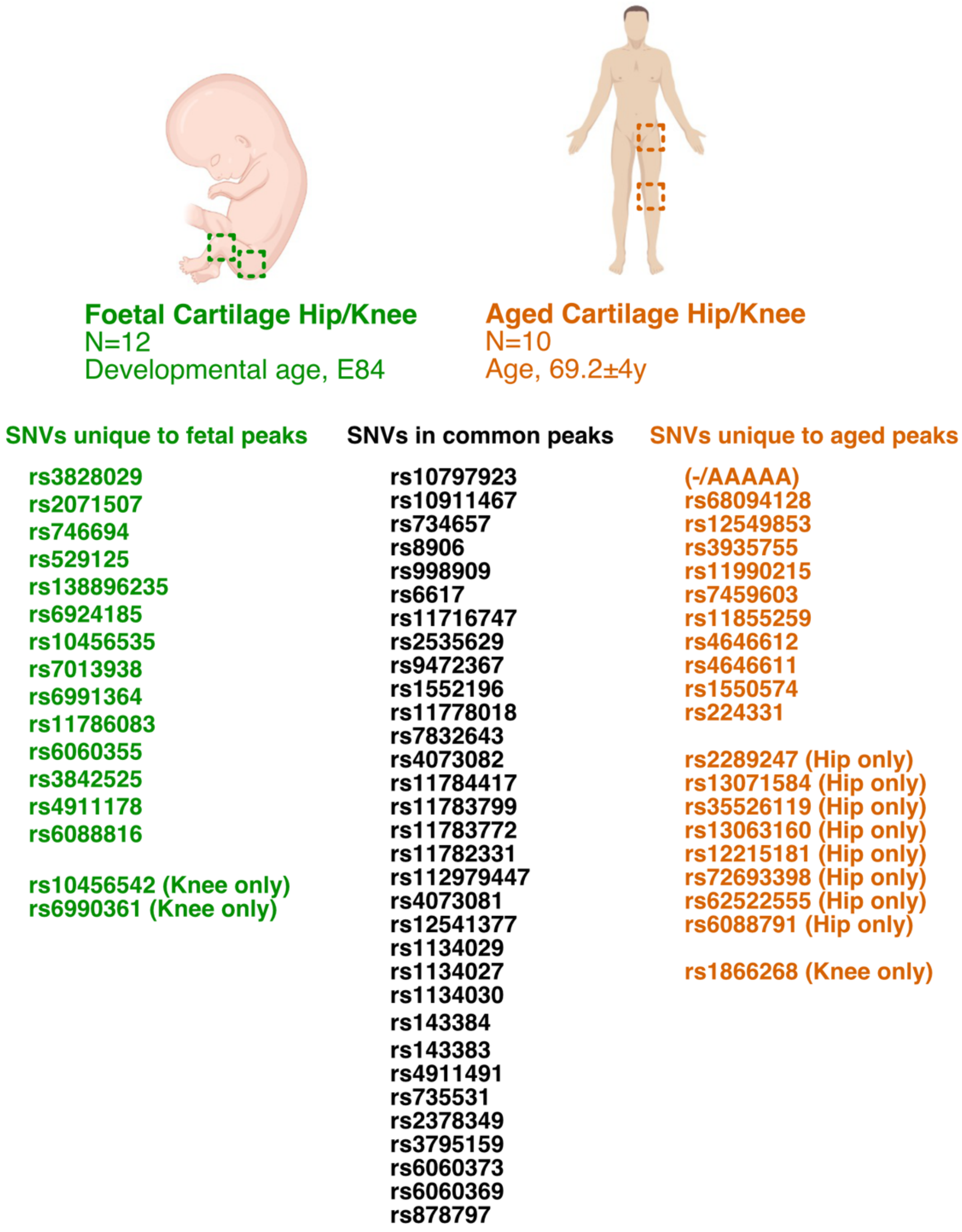

Supplementary Figure 7. SNVs that are unique to foetal and aged open chromatin regions. Upper panel, schematic diagram of human foetus and older adult indicating the joint sites from which the chondrocytes used for ATAC-seq were isolated. Lower panel, list of SNVs that uniquely intersected with open chromatin in foetal cartilage (left-hand side, green text), aged cartilage (right-hand side, orang text), and common across all four tissue types (centre, black text).
